# Detecting actionable mutations from matched plasma-based versus tissue next-generation sequencing in advanced non-small cell lung cancer: a retrospective single centre analysis on site

**DOI:** 10.1186/s13046-025-03480-x

**Published:** 2025-08-06

**Authors:** Christophe Bontoux, Caroline Lacoux, Jonathan Benzaquen, Jacques Boutros, Guylène Rignol, Elodie Long-Mira, Sandra Lassalle, Maryline Allegra, Doriane Bohly, Mathieu Garcia, Christelle Bonnetaud, Olivier Bordone, Jean-Marc Félix, Virginie Lespinet-Fabre, Virginie Tanga, Charles-Hugo Marquette, Valérie Taly, Aurélia Baurès, Simon Heeke, Marius Ilié, Véronique Hofman, Paul Hofman

**Affiliations:** 1 Department of Pathology, Cancer University Institute of Toulouse-Oncopole, University Hospital of Toulouse, Toulouse, 31059 France; 2 OncoSarc, INSERM U1037, Cancer Research Center in Toulouse, Toulouse, 31000 France; 3 Institut Hospitalo-Universitaire RespirERA, Université Côte d’Azur, Hôpital Pasteur, CHU de Nice, Nice CEDEX 1, 06001 France; 4 Laboratory of Clinical and Experimental Pathology, Université Côte d’Azur, Hôpital Pasteur, CHU de Nice, 30 Voie Romaine, Nice, 06000 France; 5 Hospital-Integrated Biobank (BB-0033-00025), Université Côte d’Azur, Hôpital Pasteur, CHU de Nice, Nice CEDEX 1, 06001 France; 6 FHU OncoAge, Université Côte d’Azur, Nice CEDEX 1, 06001 France; 7 Department of Pneumology, Université Côte d’Azur, CHU Nice, FHU OncoAge, IHU respirERA, Pasteur Hospital, Nice, France; 8 Team 4, Institute of Research on Cancer and Aging of Nice (IRCAN), Inserm U1081, CNRS UMR7284, Université Côte d’Azur, CHU de Nice, Nice CEDEX 2, 06107 France; 9 Université de Paris, UMR-S1138, CNRS SNC5096, Équipe Labélisée Ligue Nationale Contre le Cancer, Centre de Recherche des Cordeliers, Paris, France; 10 METHYS Dx, 67 rue Saint-Jacques, Paris, 75005 France; 11 Department of Thoracic/Head & Neck Medical Oncology, The University of Texas MD Anderson Cancer Center, Houston, TX USA

**Keywords:** Liquid biopsy, CtDNA, NGS, Methylation, Non-small cell lung carcinoma, Actionable genomic alterations, Precision medicine

## Abstract

**Background:**

Liquid biopsies (LB) are used increasingly to detect actionable mutations in patients newly diagnosed with advanced non-small cell lung cancer (aNSCLC), though tissue biopsies (TB) still remain the gold standard. The value of systematically combining LB and TB next-generation sequencing (NGS) for genomic profiling in these patients remains controversial.

**Methods:**

This single-centre retrospective study included 102 matched TB and LB samples collected from aNSCLC patients at diagnosis. Four circulating free DNA (cfDNA)-based NGS assays (1–4) were compared on site for performance and concordance with TB to detect ESMO Scale for Clinical Actionability of molecular Targets (ESCAT) I/II. Additionally, cfDNA droplet digital PCR methylation (ddPCR-met) testing estimated the tumour fraction to refine the interpretation of wild-type (WT) results.

**Results:**

Out of 102 patients, 13% had stage IIIB disease, and 11% presented with brain-only metastases. Adenocarcinoma was the predominant subtype (84%). Ninety LB samples yielded interpretable results across the four assays. Positive percent agreement with TB ranged from 56% (assay 2) to 79% (assay 4), with high concordance, particularly for single-nucleotide variants (SNVs). Hybrid capture-based assays (3 and 4) detected eight and seven gene fusions, respectively, while amplicon-based assays (1 and 2) detected only two each. Assay 3 only identified 12 *MET* amplifications, five of which were confirmed by fluorescence in situ hybridisation (FISH) but were missed by TB-based NGS. Five out of six negative cfDNA samples with ddPCR-met testing were WT across all assays. The plasma-first approach added incremental value, up to 21% (assay 3). Amplicon-based assays were faster and required less input of DNA for analysis. Patients with stage IIIB or brain-only metastases were significantly more likely to have negative/low levels of cfDNA ddPCR-met.

**Conclusions:**

LB-based NGS demonstrated high concordance with TB in newly diagnosed aNSCLC, particularly for detection of SNV. Hybrid capture assays showed superior performance in identifying gene fusions and *MET* amplifications. The incremental value of a plasma-first strategy was limited in this real-life study. Thus, LB-based NGS on site should be seen as a complementary tool to TB-based NGS or an alternative when tissue samples are unavailable. Additionally, cfDNA methylation analysis enhances diagnostic accuracy in specific cases.

**Supplementary Information:**

The online version contains supplementary material available at 10.1186/s13046-025-03480-x.

## Introduction

Management of non-small cell lung cancer (NSCLC) is a major challenge in clinical oncology, accounting for the highest proportion of cancer-related deaths worldwide [1]. Despite advancements in therapeutic approaches, improving the outcome of NSCLC patients hinges on the timely identification of actionable genetic alterations and the implementation of targeted therapies [2]. The European Society for Medical Oncology (ESMO) recommends using next-generation sequencing (NGS) assays for screening specific genetic alterations classified under the ESMO Scale for Clinical Actionability of molecular Targets (ESCAT) in tumour tissue biopsies (TB) [2, 3]. ESMO ESCAT I/II genomic alterations involving the *EGFR*, *ALK*, *KRAS*, *ROS1*, *BRAF*, *MET*, *ERBB2*, *NRG1*, *FGFR1/2/3*, *RET* and *NTRK1/2/3* genes have the strongest evidence supporting their use in guiding targeted therapies in advanced (a) NSCLC [2, 4, 5]. Identifying these alterations is essential for patient stratification and advances in precision oncology [3].

Recent advances in LB have revolutionised the field of molecular diagnostics and therapeutic decision-making for aNSCLC [6]. Among the various LB components, circulating tumour DNA (ctDNA) has emerged as a highly promising biomarker. ctDNA offers valuable insight into a tumour’s genetic landscape by enabling the detection of actionable mutations, treatment responses, resistance mechanisms and real-time tumour dynamics [7, 8].

Compared to the traditional gold standard TB, which is invasive and time-consuming, LB provides a minimally invasive and repeatable approach. It facilitates quicker turnaround times for genotyping and can serve as an alternative or complementary tool to TB, particularly for patients with aNSCLC. Its utility is critical in scenarios where tissue samples are insufficient in routine clinical practice (15 to 40% of cases), a procedure is not feasible, or the specimen cannot meet the urgent timeline required for treatment decisions [9–13]. However, international guidelines currently recommend the use of LB in aNSCLC at diagnosis only under specific conditions, such as a low-quality, unavailable/not accessible or inadequate tissue samples [11].

The introduction of NGS has been pivotal in unraveling the complex genomic landscape of NSCLC. NGS enables comprehensive profiling by identifying key driver mutations, including ESCAT I/II gene alterations [3, 14–17]. This innovation has laid the foundations for personalised medicine, allowing for highly tailored treatments based on a tumour’s molecular profile. Despite its advantages, implementing NGS in a ctDNA analysis on site nowadays remains technically challenging. The associated biological challenge includes the low abundance and fragmented nature of ctDNA molecules in circulation, which can often lead to non-informative results. This could be perceived as discouraging by many physicians [18].

Questions persist regarding the concordance of results between LB and TB and whether integrating both methods into daily practice, notably at tumour diagnosis, offers clear clinical advantages (i.e. turnaround time to treatment and/or detection rate of actionable alterations) [13, 19–26]. However, when both TB and LB-based NGS are used synergistically, they may provide a broader perspective into the tumour biology, supporting more comprehensive diagnostics. Another critical aspect influencing the reliability of LB is the accurate evaluation of the circulating tumour DNA fraction (TF). This helps differentiate between false-negative and true-negative results. Emerging techniques such as analysing copy number variations (CNVs), fragmentomic approaches, specific mutations and methylation profiles are increasingly being adopted to improve the diagnostic accuracy of ctDNA analyses [27–29].

We conducted a single-centre and retrospective study to evaluate the feasibility and clinical relevance of routinely performing matched TB and LB-based NGS analyses onsite for patients at diagnosis of aNSCLC. The study aimed to demonstrate how both approaches complement each other in identifying clinically significant ESMO ESCAT I/II genetic alterations. Additionally, we investigated the circulating free DNA (cfDNA) methylation status to provide insight into estimation of the TF and integrate the findings of cfDNA into broader molecular profiles, highlighting the prognostic significance of ctDNA in aNSCLC.

## Methods

### Selection of cases

**Fig. 1:**
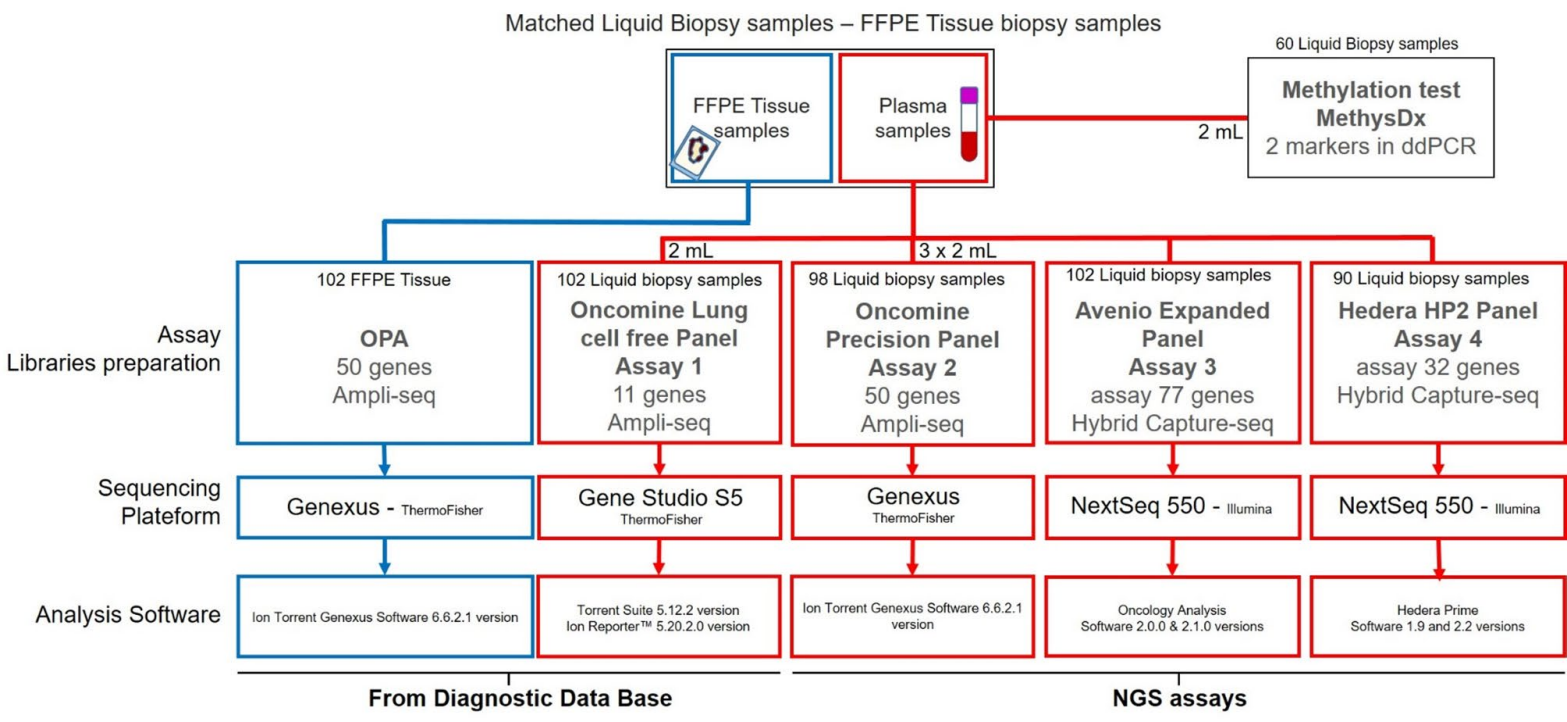
Flowchart depicting all assays used on patients’ samples (TB and LB samples)

Cases were selected according to the quality of the TB NGS analyses and if enough blood of matched liquid biopsies was available in order to process the four LB NGS assays from the same sample. Moreover, patients were included in this study only in the case of advanced stage NSCLC. The study design of the four assays evaluated is depicted in Fig. 1.

### TB cfDNA/RNA extractions and NGS assay

As previously described [30, 31], DNA and RNA were independently extracted from formalin-fixed paraffin-embedded (FFPE) samples, including biopsies, cytological specimens and surgically resected specimens, using the Maxwell RSC Instrument (Promega, #AS4500) with the Maxwell RSC FFPE Plus DNA Kit (#AS1720) and Maxwell RSC RNA FFPE Kit (#AS1440). Extracted nucleic acids were stored at -80°C to preserve integrity. NGS was performed using the Oncomine™ Precision Assay GX (Thermo Fisher Scientific, #A46291). This assay targets 50 key genes, including 45 for DNA mutation analysis, 18 for fusion detection, and 14 for CNV identification. Additionally, it incorporates a 5’/3’ expression imbalance caller for identifying novel fusions.

### LB circulating and total nucleic acid extraction

For LB collection, 18 mL of blood was drawn into EDTA (Greiner BIO ONE, Kremsmünster, Austria, #455036) or Streck tubes (Madison Industries Holdings LLC, La Vista, NE, USA) and processed within 2 h. Samples were first centrifuged at 2000 x g for 10 min at 4 °C [32]. Plasma was separated into new tubes, centrifuged at 5000 x g for 10 min at 4 °C, and stored in 1 mL aliquots at -80 °C.

cfDNA and circulating RNA (cfRNA) were extracted from 8 mL of plasma using the QIAamp Circulating Nucleic Acid Kit (#55114) in accordance with the manufacturer’s protocol. The concentrations of total cell-free nucleic acids (cfTNA) (ng/µL) were measured using the Qubit Fluorometer with the Qubit dsDNA HS Assay Kit (#Q32851). Extracted nucleic acid was stored at -80 °C and gently thawed on ice prior to library preparation.

### LB NGS assays

Four gene assays were used to detect gene alterations in cfDNA/cfRNA. Amplicon-based NGS was employed using two focused assays (Assays 1 and 2) designed for specific target amplification. Hybrid capture-based NGS was applied through two comprehensive assays (Assays 3 and 4), using probes to capture target sequences across a broader genomic scope. The workflows of all LB-based assays are depicted in Supplementary figure S1. Descriptions of all assays can be found in the Supplementary information - Materials and Methods.

*Assay 1*: The Oncomine Lung cfTNA panel (Thermo Fisher Scientific, Waltham, USA, #A35864) targets 11 key genes. It includes 11 genes for single-nucleotide variant (SNV) detection, covering 168 hotspots, CNV detection in the *MET* gene and fusion detection in *AL*K, *RET*, and *ROS1* genes [31, 33]. This panel required starting material for cell-free nucleic acids ranging from 1.3 ng to 146 ng, with a median of 7.7 ng (5 to 20 ng corresponding to the supplier’s recommendations). Libraries were prepared using the Ion Chef instrument, and sequencing was performed on the Ion S5 system. Variant calling relied on the S5 Torrent Server, analysed using Ion Reporter software. For this assay the Limit of Detection was 0.1% with a minimal total and molecular depth of 25 000 and 2 500, respectively. The minimum read number was 8 reads for fusions and 3 reads for hot spots. One positive control (Structural Multiplex cfDNA Reference Standard HD786, Horizon Discovery, Waterbeach, UK) was used for each run as part of the diagnostic routine workflow. Negative controls (WT samples) were used as part of intra-laboratory diagnostic quality control workflow.

*Assay 2*: The Oncomine Precision Assay GX panel (Thermo Fisher Scientific, #A46291) targets 50 key genes, including 45 for SNVs, 14 for CNVs, and 18 for fusions [31]. This panel required starting material amounts between 2.5 ng and 46.5 ng, with a median of 14.5 ng (from 1 to 50 ng according to the supplier’s recommendations). The assay utilised the Ion Torrent Genexus system (Thermo Fisher Scientific), which automates library preparation, templating, sequencing and analysis of NGS results, ensuring streamlined processing and reporting. For this assay, the minimum variant allele frequency (VAF) was 0.5%. No Limit of Detection was provided by the software. The minimal read number was 20 reads for fusions and 5 reads for hot spots. The minimal total and molecular depth were 25 000 and 500, respectively with recommended ranges of 22 000–40 000 and 1000–3000, respectively. No batch-level positive/negative controls were used for these experiments.

*Assay 3*: The Avenio Expanded panel (F. Hoffmann-La Roche, Bâle, Switzerland) targets 77 genes, including SNVs across all 77 genes, CNVs in 3 genes and 6 fusion genes [34, 35]. The panel covers a total region size of 200 kb, ensuring complete or specific exon coverage for each gene. Starting cfDNA input ranged from 2.5 ng to 50.4 ng, with a median of 25.8 ng (range recommended by suppliers: 10 to 50 ng). Library preparation began with overnight barcoding (~ 16 h) using unique molecular identifiers (UMIs) and sample-specific adapters (mono-adapters with unique barcodes). Next, libraries were amplified by polymerase chain reaction (PCR) and subsequently enriched via hybridisation with specific avidin coupled-probes for 16 h. Captured library complexes were isolated using streptavidin beads, followed by stringent washing to remove off-target sequences. The enriched libraries were amplified and cleaned-up again, and their quality assessed using the Agilent Bioanalyzer 2100 (Agilent Technologies, Santa Clara, CA, USA) system with DNA High Sensitivity chips (#s 5067 − 4626 and 5067 − 4627). Final libraries were pooled in equimolar ratios and sequenced on the Illumina NextSeq 500 or NextSeq 550Dx platforms (Illumina Inc., San Diego, CA, USA). Sequencing data were analysed using Roche’s Oncology Analyst Software for comprehensive result generation. The minimal VAF was 0.5% according to a limit of detection of 25 alternative unique reads on 5000 unique total reads. A single commercial positive control (Structural Multiplex cfDNA Reference Standard HD786, Horizon Discovery, Waterbeach, UK) was used during the first analytical run.

*Assay 4*: The Hedera Profiling 2 ctDNA test panel (Hedera Dx, Epalinges, Switzerland) targets 32 genes (SNVs), 15 genes (CNVs), 8 fusion genes and 36 microsatellite instability (MSI) markers [36]. The panel spans a region size of 90 kb, covering whole exon regions of 19 of the targeted genes. cfDNA input ranged from 3 ng to 30 ng, with a median of 17 ng (minimum of 10 ng according to the supplier’s recommendations). Libraries were prepared following a procedure similar to the Avenio panel (Assay 3), starting with UMI indexing and dual-index barcoding. Precapture libraries underwent quality and quantity checks to create an equimolar pool of sublibraries. The pooled libraries were hybridised overnight (~ 16 h) with panel-specific avidin coupled-probes, followed by capture on streptavidin beads. Amplification of the enriched libraries was performed via PCR, and the final pools were quality-checked using the Agilent Bioanalyzer 2100 system with High Sensitivity DNA chips. Sequencing was conducted on the Illumina NextSeq 500 or NextSeq 550Dx systems following manufacturer instructions. Data from this panel were processed using the Hedera Prime pipeline to deliver comprehensive sequencing results. The minimal VAF was 0.5% corresponding to 2 alternative unique duplex read limits of detection (High confidence alteration detection). Under 2 alternative unique duplex read variants are considered as low confidence. A single positive commercial control (5 Gene Multiplex 5% AF cfDNA, Goffin Molecular Technologies, Beesd, NL) was used during the first analytical run.

### cfDNA extraction and analysis by methylation-specific droplet digital PCR (ddPCR-met)

The cfDNA was extracted from plasma using the QIAamp^®^ Circulating Nucleic Acid Kit (QIAGEN, #55114) according to the manufacturer’s instructions, with a final elution volume of 50 µL (AVE buffer). Incubation with proteinase K was performed for 30 min at 60 °C. Each DNA extraction batch included a no cfDNA sample consisting of phosphate-buffered saline 1x buffer (PBS). Met-ddPCR was performed by METHYS Dx using proprietary developed assays and procedures (#EP3945135-A1, #WO2022023233-A1, #WO2022023233-A9) [37]. DNA was quantified with the Qubit™ dsDNA HS kit (Invitrogen™, #Q32854).

Bisulfite conversion of DNA samples was done with the EZ DNA Methylation-Gold Kit (Zymo Research, #ZD5006) or the EZ DNA Methylation-Lightning Kit (Zymo Research, #ZD5031) as recommended by the manufacturer. Twenty ng was used when possible or 20 µL from low-concentration samples.

For each conversion, two controls were performed: (1) a negative control (10 ng of human genomic DNA extracted from whole blood pooled from multiple donors, Promega^®^, #G304A), and (2) a positive control (10 ng of enzymatically methylated human genomic DNA, Zymo Research, #D5011). Plasma samples were screened for the presence of methylation markers by ddPCR-met, as previously described [38], using Bio-Rad QX200 assay (QX200 Generator, PX1 Sealer, Thermocyclers C1000/S1000, and QX200 Reader).

This approach allowed us to determine the presence or absence of ctDNA and its quantity (ng/mL of plasma). Analysis was performed using a triplex assay targeting two methylated cancer-specific genes (Homeobox B4 (*HOXB4*) and Maestro heat-like repeat family member 6 (*MROH6*)), with a methylation-insensitive target on the albumin (*ALB*) gene used as reference. For each assay, LOB (Limit Of Blank) and LOD (Limit Of Detection) were calculated as previously described [39]. Data processing was carried out with Quantasoft Analysis Pro (BIORAD, USA) v1.0 software. Patient samples with a number of copies/20µL higher than LOB were considered positive for ctDNA testing, while patients with a number of copies/20µL lower than LOB and with 200 copies/20µL *ALB* minimum were considered negative. Samples of patients with a number of copies/20µL between the LOB and the LOD were considered as positive borderline. Samples of patients with a number of *ALB* copies lower than 200 copies/20µL were considered non-interpretable.

### MET FISH analysis

*MET* FISH analysis was carried out using the Vysis *MET* SpectrumRed/Centromere enumeration probe (CEP) 7 SpectrumGreen FISH Probe Kit (Vysis, Abbott Molecular). Tissue sections, 3 μm thick, were prepared for FISH staining; the process was performed according to the manufacturer’s instructions. For analyses, 60 nuclei were scored for signals using a Nikon, Eclipse 80i microscope equipped with a triple-pass filter (DAPI/Green/Orange, Vysis), at a final magnification of 600×. *MET* gene status was classified according to Schildhaus et al. and the Acsé French NCI program [40, 41]. Amplification was defined as one of the following aspect: (1) *MET*/CEN7 ratio ≥ 2.0 or (2) average gene copy number ≥ 6.0 per tumor cell or 3) ≥ 10% of tumor cells containing ≥ 15 *MET* signals.

### Statistical analysis (Comparison of the outcome of the LB and TB assays)

This study evaluated amplicon-based and hybrid capture-based NGS strategies for LB analysis [30]. The amplicon-based NGS method involves the use of PCR to amplify regions of interest (ROI). PCR amplicons are barcoded, further amplified and prepared using Ion Torrent NGS technology (Thermo Fisher Scientific, Waltham, MA, USA) [42]. Conversely, the hybrid capture-based NGS method incorporates a hybridisation step, where probes from commercial panels anneal to ROI, followed by a capture process using affinity beads. Libraries were amplified via PCR both before barcoding and after hybridisation/capture reactions. This technique is performed using Sequencing-By-Synthesis Illumina NGS technology from Illumina (Illumina Inc., San Diego, CA, USA) [43].

The turnaround time, defined as the period from plasma collection to generation of the results using manufacturer-specified analysis software, includes extraction, library preparation (manual or automated), sequencing (Ion Torrent and Illumina technologies), as well as primary, secondary and post-analytical stages. Amplicon-based strategies are generally faster and less labour-intensive, as they avoid the prolonged hybridisation incubation (16–18 h) and stringent washing steps required in hybrid capture methods.

Comparison between the outcomes of different LB panels was performed to assess their relative efficiencies. To assess the diagnostic accuracy of LB, the TB results were used as the reference, with analysis focusing on actionable ESCAT I/II genomic alterations by comparing the findings of the LB panel with the corresponding TB results for each patient. ESMO ESCAT Tier I/II includes SNVs in *EGFR*, *KRAS*,* ERBB2* and *BRAF genes*; *MET* CNVs; *MET* exon 14 skipping; and fusions involving *ALK*, *NRG1*,* FGFR1/2/3*,* ROS1*,* RET and NTRK1/2/3* [3]. The data were further analysed to explore the potential of plasma-first reflex testing as an alternative to tissue-first testing. The “Liquid-first incremental value” refers to the proportion of biomarker-positive patients who would have not been identified without complementary tissue NGS. In contrast, the “Tissue-first incremental value” refers to biomarker-positive patients rescued via liquid NGS.

Tissue gene alterations were categorised into two groups: Mutant, encompassing ESCAT I/II alterations, and WT, including ESCAT III/IV/V/X alterations and WT.

Diagnostic metrics were calculated for each LB panel, including (1) positive percent agreement (PPA), defined as the number of mutants in LB divided by the sum of mutants and WT in LB for TB mutants; (2) negative percent agreement (NPA), calculated as the number of WT in LB divided by the sum of WT and mutants in LB for TB WT; (3) accuracy, defined as the sum of real mutants (in both LB and TB) and real WT (in both LB and TB) divided by the total number of alterations; (4) positive predictive value (PPV), calculated as the number of mutants in LB for TB mutants divided by the total number of mutants in LB for both TB mutants and TB WT; and (5) negative predictive value (NPV), defined as the number of WT in LB for TB WT divided by the total number of WT in LB for both TB WT and TB mutants.

Discordant cases were defined as those in which all cfDNA NGS assays failed to detect alterations identified with TB NGS.

Data are presented as medians for continuous variables and percentages for categorical variables. Fisher’s exact test was used to compare categorical variables, and Student’s t-test was used to compare means or medians. The Kaplan-Meier method with a log-rank test was used to compare overall survival curves. All analyses were performed using R software (including “Stataid” R package) [44].

## Results

### Patient characteristics

The baseline characteristics and gene alterations of the 102 aNSCLC patients included in this study are summarised in Table 1. The median age at diagnosis was 65 years (range 56–73 years), with a near-equal gender distribution. Most patients (87%) presented with stage IV disease. Histologically, the majority of tumours were adenocarcinomas (82%) based on WHO 2021 classification [45]. Regarding testing workflows, 63% of patients underwent TB testing first, with a median delay of 6.5 days, compared to 21% tested via LB first, with a shorter median delay of 1 day. Simultaneous LB and TB testing was conducted in 16% of cases. The median follow-up was 5.5 months (range: 0.25–11 months). Treatment decision-making was based on both the LB and TB NGS results in 61% of patients, on TB results alone in 37%, and on LB results alone in 2%. Supplementary figure S2 shows the distribution of all gene alterations detected in patient samples at baseline in each assay. *TP53* SNVs were the most prevalent alteration detected in our cohort in both TB and LB samples.

**Table 1:** Baseline patient characteristics and gene alterations

VARIABLE	Whole cohort
**CLINICAL DATA**	
**Age at diagnosis, y**	
Median [IQR]	65 [56–73]
**Gender**	
F	52 (51)
M	50 (49)
**Stage**,** n (%)**	
IIIB	13 (13)
IV	89 (87)
**Primary Tumour Size (mm)**	
Median [IQR]	60 [35.5–73.5]
**Metastasis location**,** n (%)**	
Locoregional	21 (26)
Brain only	11 (14)
Distant	48 (60)
**Single brain metastasis**,** n (%)**	
Yes	11 (12)
No	79 (88)
**HISTO-MOLECULAR DATA**	
**WHO 2021 histological subtype**,** n (%)**	
Adenocarcinoma	84 (82)
Squamous cell carcinoma	5 (5)
LCNEC	1 (1)
NSCLC, NOS	12 (12)
**TB and LB NGS testing**	
LB first	22 (21)
Delay, Median [IQR], d	1 [0-11.5]
TB first	64 (63)
Delay, Median [IQR], d	6.5 [1.75–17.25]
Same time	16 (16)
***HOXB4 *** **marker cfDNA ddPCR-met (copies/20µL)**	
Mean (sd)	3.41 (6.12)
Median [IQR]	0.75 [0.08–3.45]
***MROH6 *** **marker cfDNA ddPCR-met (copies/20µL)**	
Mean (sd)	6.25 (14.55)
Median [IQR]	0.65 [0-4.9]
**cfDNA ddPCR-met status**	
Negative	12 (20)
Positive	37 (61.67)
Positive borderline	11 (18.33)
**DISTRIBUTION OF ESCAT I/II GENE ALTERATIONS IN TB**	
***EGFR *** **alterations**	15 (15)
Common mutation (del19, L858R)	10 (10)
Exon 20 insertions	1 (1)
Uncommon mutations (G719, variant in exon 18, L861Q, S768I)	4 (4)
***KRAS *** **G12C**	9 (9)
***BRAF *** **V600E**	2 (2)
***ERBB2 *** **hotspot mutations**	3 (3)
***ALK *** **alterations**	8 (8)
Fusion	7 (7)
Hotspot acquired mutation	1 (1)
***MET *** **mutations ex14 skipping**	5 (5)
***NTRK *** **fusion**	1 (1)
***FGFR *** **fusion**	1 (1)
**FOLLOW-UP DATA**	
**Follow-up**,** m**	
Median [IQR]	5.5 [0.25-11]
**Treatment based on**,** n (%)**	
LB and TB NGS results	62 (61)
LB NGS results	2 (2)
TB NGS results	37 (37)

cfDNA, circulating free DNA; ddPCR-met, droplet digital PCR methylation; ESCAT, ESMO Scale for Clinical Actionability of molecular Targets) I/II levels; IQR, interquartile range; LB, liquid biopsy; LCNEC, large-cell neuroendocrine carcinoma; NGS, next-generation sequencing; NOS, not otherwise specified; NSCLC, non-small cell lung cancer; SD, standard deviation; TB, tissue biopsy

### Concordance and performance of TB and LB-based NGS panels in detecting key gene alterations and the impact of plasma-first screening

**Fig. 2:**
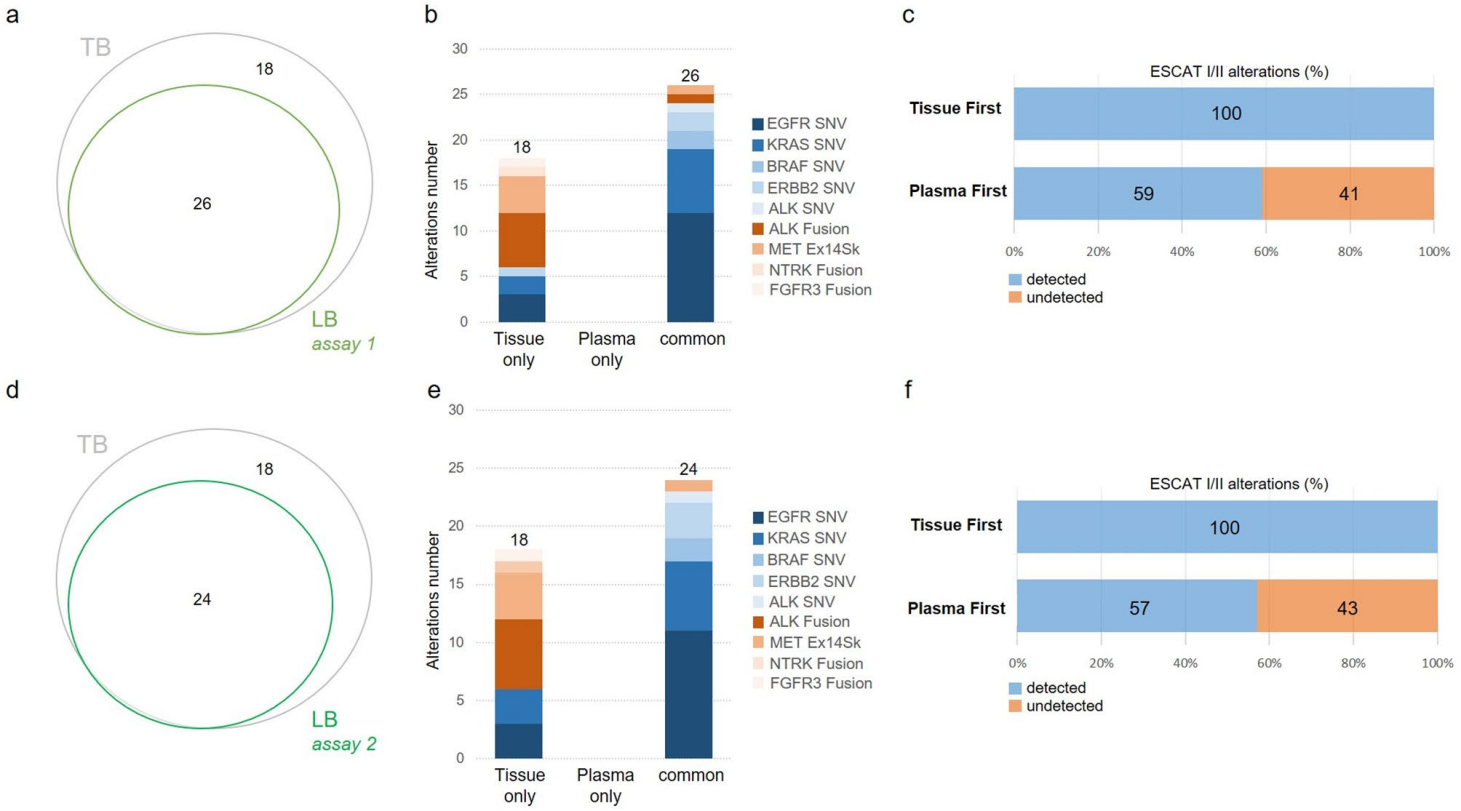
Comparison between ESCAT I/II alterations detected in TB and matched LB samples using amplicon-based cfDNA assays. Assay 1 (*n* = 102): (**a**) Venn diagram depicting the proportion of actionable alterations detected in TB and LB, (**b**) Bar plot representation of alterations distribution in TB only, LB only and both (concordance), (**c**) Frequency of activable alterations detected by reflex testing using TB or LB first. Assay 2 (*n* = 98): (**d**) Venn diagram depicting proportion of actionable alterations detected in TB and LB, (**e**) Bar plot representation of alterations distribution in TB only, LB only and both (concordance), (**f**) Frequency of activable alterations detected by reflex testing using first TB or LB

Variants detected using the amplicon-based cfDNA panel assays (Assays 1 and 2) categorised by ESCAT I/II guidelines are shown in Fig. 2. For Assay 1, 44/102 patients (43.1%) had gene alterations identified in their samples by either TB or LB, 26 of which had gene alterations identified by both assays (26/44; 59%) and 18 of which had gene alterations detected only in TB (18/44; 41%) (Fig. 2a). 92% (24/26) of gene alterations detected in both TB and LB were SNVs while two gene fusions out of 14 were detected in LB (Fig. 2b). For Assay 2, 42/96 patients (43.8%) had gene alterations identified in their samples by either TB or LB, 24 of which had mutations identified by both assays (24/42; 57%) and 18 of which had gene alterations detected only in TB (18/42; 43%) (Fig. 2d). 96% (23/24) of gene alterations detected in both TB and LB NGS were SNVs while one gene fusion out of 14 was detected in LB (Fig. 2e).

**Fig. 3:**
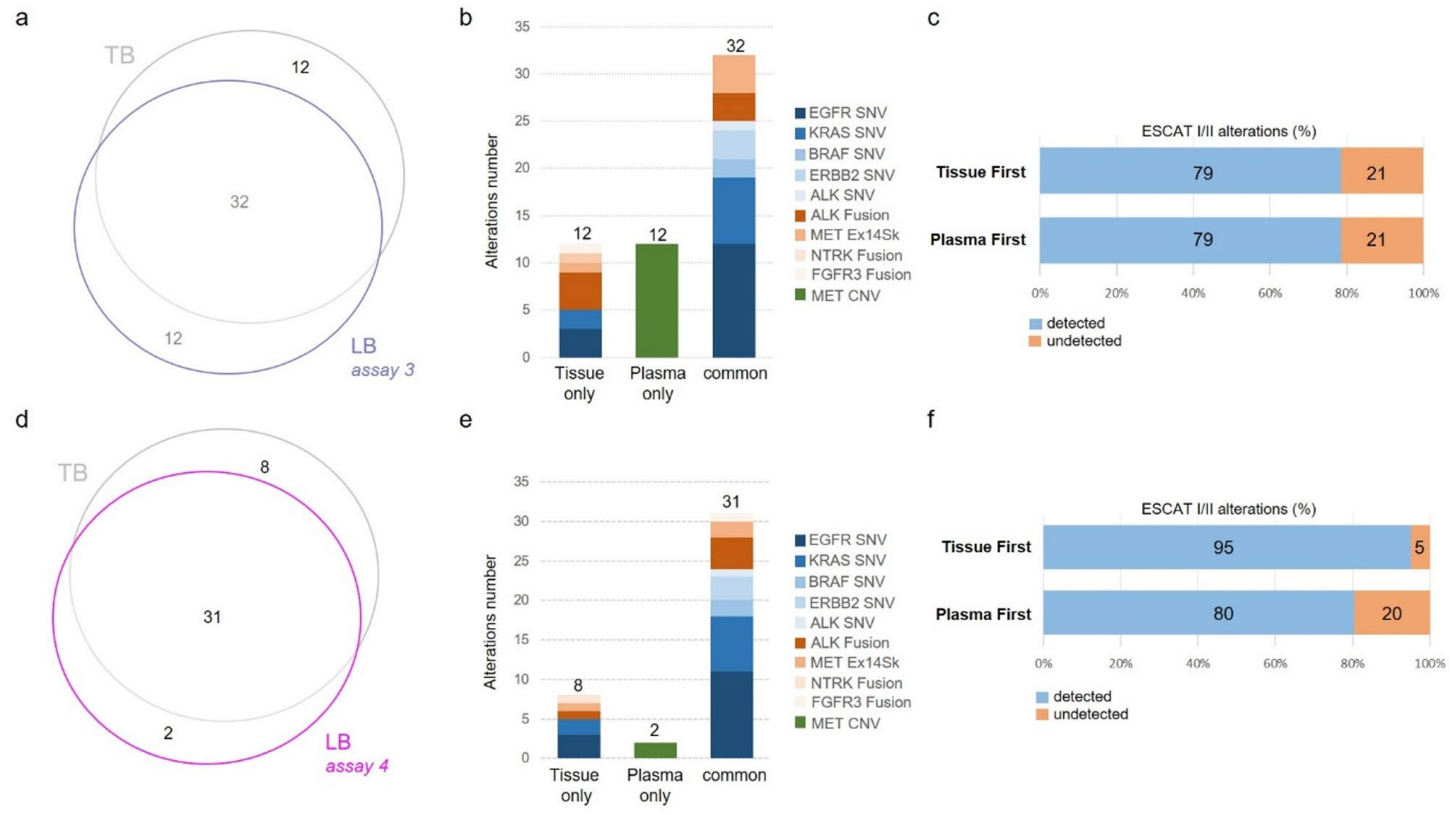
Comparison between ESCAT I/II alterations detected with TB and matched LB samples using hybrid capture-based cfDNA assays. Assay 3 (*n* = 102): (**a**) Venn diagram depicting the proportion of actionable alterations detected in TB and LB, (**b**) Bar plot representation of alterations distribution in TB only, LB only and both (concordance), (**c**) Frequency of activable alterations detected by reflex testing using TB or LB first. Assay 4 (*n* = 90): (**d**) Venn diagram depicting the proportion of actionable alterations detected in TB and LB, (**e**) Bar plot representation of the distribution of alterations in TB only, LB only and both (concordance), (**f**) Frequency of activable alterations detected by reflex testing using first TB or LB

Variants detected using the hybrid capture-based cfDNA panel assays (Assays 3 and 4) categorised by ESCAT I/II guidelines are shown in Fig. 3. For Assay 3, 56/102 patients (54.9%) had gene alterations identified in their samples by either TB or LB, 32 of which had gene alterations identified by both assays (32/56; 57%) and 12 of which had gene alterations detected only in TB (12/56; 21%) (Fig. 3a). 78% (25/32) of gene alterations detected in both TB and LB NGS were SNVs while seven gene fusions out of 14 were detected in LB (Fig. 3b). For Assay 4, 41/90 patients (45.6%) had gene alterations identified in their samples by either TB or LB, 31 of which had mutations identified by both assays (31/41; 76%) and 8 of which had gene alterations detected only in TB (8/41; 20%) (Fig. 3d). 77% (24/31) of gene alterations detected in both TB and LB NGS were SNVs while seven gene fusions out of 14 were detected in LB (Fig. 3e). Hybrid capture-based LB NGS assays detected 12 (Assay 3) and two (Assay 4) *MET* CNVs that were not detected by TB-based NGS (Fig. 3b and e). All assays showed high concordance with TB NGS and among themselves for detecting ESMO ESCAT I/II gene mutations (SNVs). However, concordance for gene fusion and *MET* amplification was lower (Fig. 2b and e and Fig. 3b and e). Capture-based panels (Assays 3 and 4) performed better for detecting *MET* CNVs. Specifically, Assay 3 was more effective in detecting *MET* CNVs (Fig. 3b).

Overall, the liquid-first incremental value varied from 20 to 43%, depending on the LB-based NGS assay, versus 0–21% for the tissue-first incremental value (Fig. 2c and f and Fig. 3c and f).

### Variations in LB-based panel efficiencies and detection rates

**Fig. 4:**
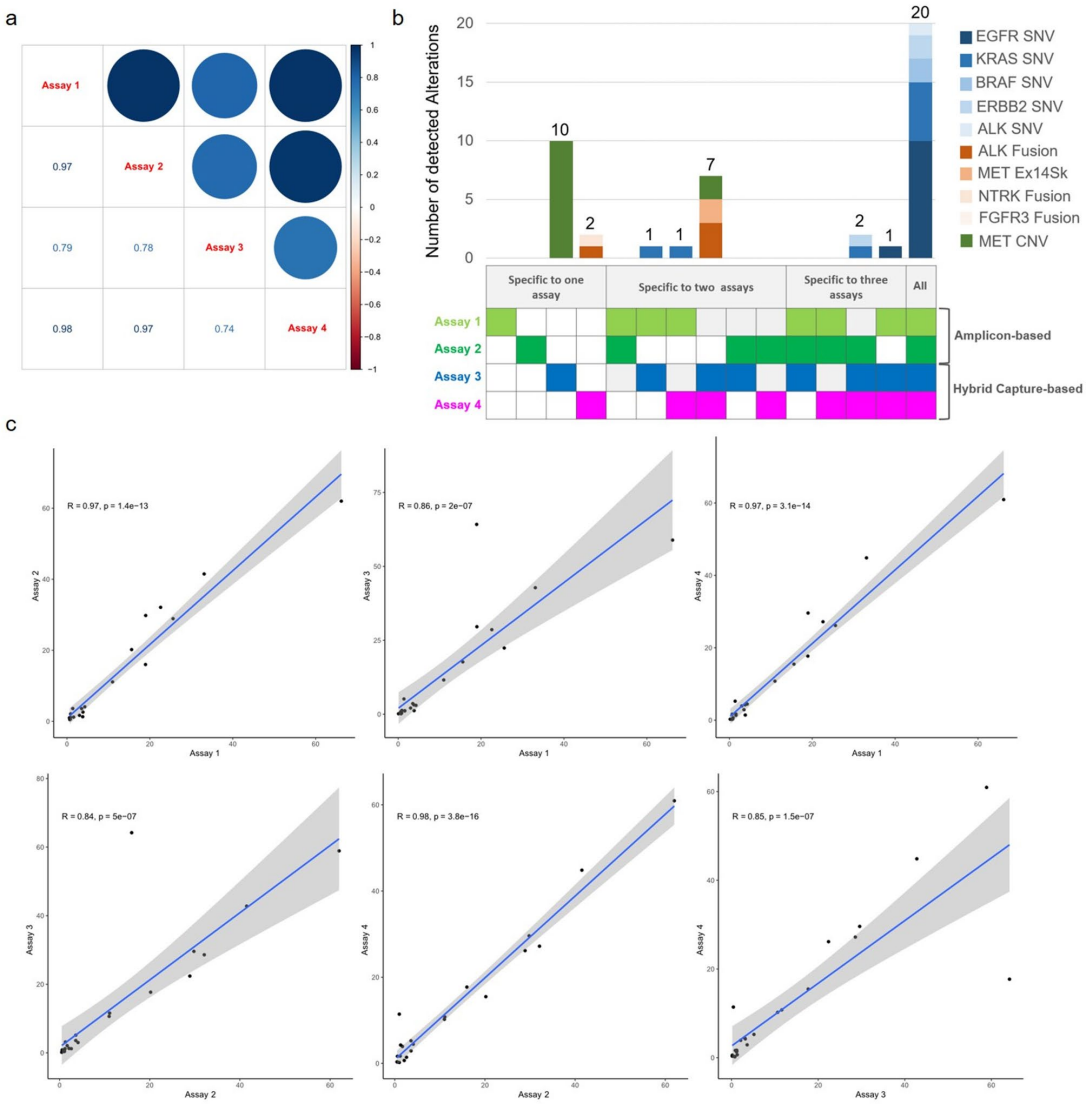
Comparison between ESCAT I/II alterations detected in plasma using hybrid capture-based assays and amplicon-based assays: (**a**) Correlation matrix depicting pairwise relationships between the assays for ESCAT I/II mutations. The strength and direction of correlations are represented by the colour intensity and hue, respectively, according to the provided scale bar as well as the numerical value provided on the left. (**b**) Bar plot representation of alterations detected specifically in one, two or three assays for both amplicon and hybrid capture-based methods. (**c**) Correlation of VAF for ESCATI/II mutations across all panels. The coefficient of determination (R) as well as *p*-value are shown

Overall, 90 LB samples gave interpretable results with all four cfDNA NGS assays. Each assay showed high concordance with the TB NGS results and among themselves for detecting gene alterations, notably for SNV detection (100% of gene alterations detected in all LB NGS assays were SNVs) (Table 2 and Fig. 4). Correlation matrix showed pairwise relationships between the assays for ESCAT I/II SNV detection ranging from 0.84 to 0.98 (Fig. 4a). The PPA and NPA of each of the four LB-based NGS assays compared with TB NGS ranged from 56% (Assay 2) to 79% (Assay 4) and 80% (Assay 3) to 100% (Assay 1 and 2), respectively (Table 2). Overall, Assay 4 demonstrated the highest accuracy (89%) and PPA (79%), making it the most effective among the four LB-based assays for detecting ESCAT I/II gene alterations.

**Table 2:** Performance of each panel assay tested in the study for ESCAT I/II gene alteration detection

ESCAT I/II alterations (Patients, *N* = 102)			Assay 1	Assay 2	Assay 3	Assay 4
**mut**	43	**mutated**	26	23	31	30
		**WT**	17	18	12	8
**WT**	59	**mutated**	0	0	12	2
		**WT**	59	55	47	50
		**Total patients (n)**	**102**	**96**	**102**	**90**
		**PPA**	60%	56%	72%	79%
		**NPA**	100%	100%	80%	96%
		**Accuracy**	83%	81%	77%	89%
		**PPV**	100%	100%	72%	94%
		**NPV**	78%	75%	80%	86%

PPA, positive percent agreement; mut, mutated; NPA, negative percent agreement; PPV, positive predicting value; NPV, negative predicting value; WT, wild-type

A comparison of outcomes across various LB panels was conducted to evaluate their respective efficiencies. The agreement for gene fusions and *MET* amplifications among these assays was lower compared to SNVs (Fig. 4b). Hybrid capture-based assays were more effective than amplicon-based assays in detecting gene fusions and *MET* amplifications, particularly when the ctDNA input exceeded 30 ng (Fig. 4b and Supplementary Figure S3). Overall, Assay 4 showed slightly better performance in identifying gene fusions, while Assay 3 was better at detecting *MET* amplifications. 10 *MET* CNVs were detected only in Assay 3 while two gene fusions (one *ALK* and one *FGFR3* fusions) were detected only in Assay 4 (Fig. 4b). Significant values of the correlation of VAF for ESCAT I/II mutations across all panels were also observed (Fig. 4c). Finally, Supplementary Figure S4 highlights how VAF influenced concordance among cfDNA NGS assays. Six out of seven discordant cases for ESCAT I/II SNV detection showed a VAF below or around 1%. One *HER2* L755P SNV was found by assays 2, 3 and 4 with a VAF higher that 10%, however, assay 1 did not cover this SNV.

### cfDNA methylation biomarker analysis and its role in enhancing the accuracy of liquid biopsy

**Fig. 5:**
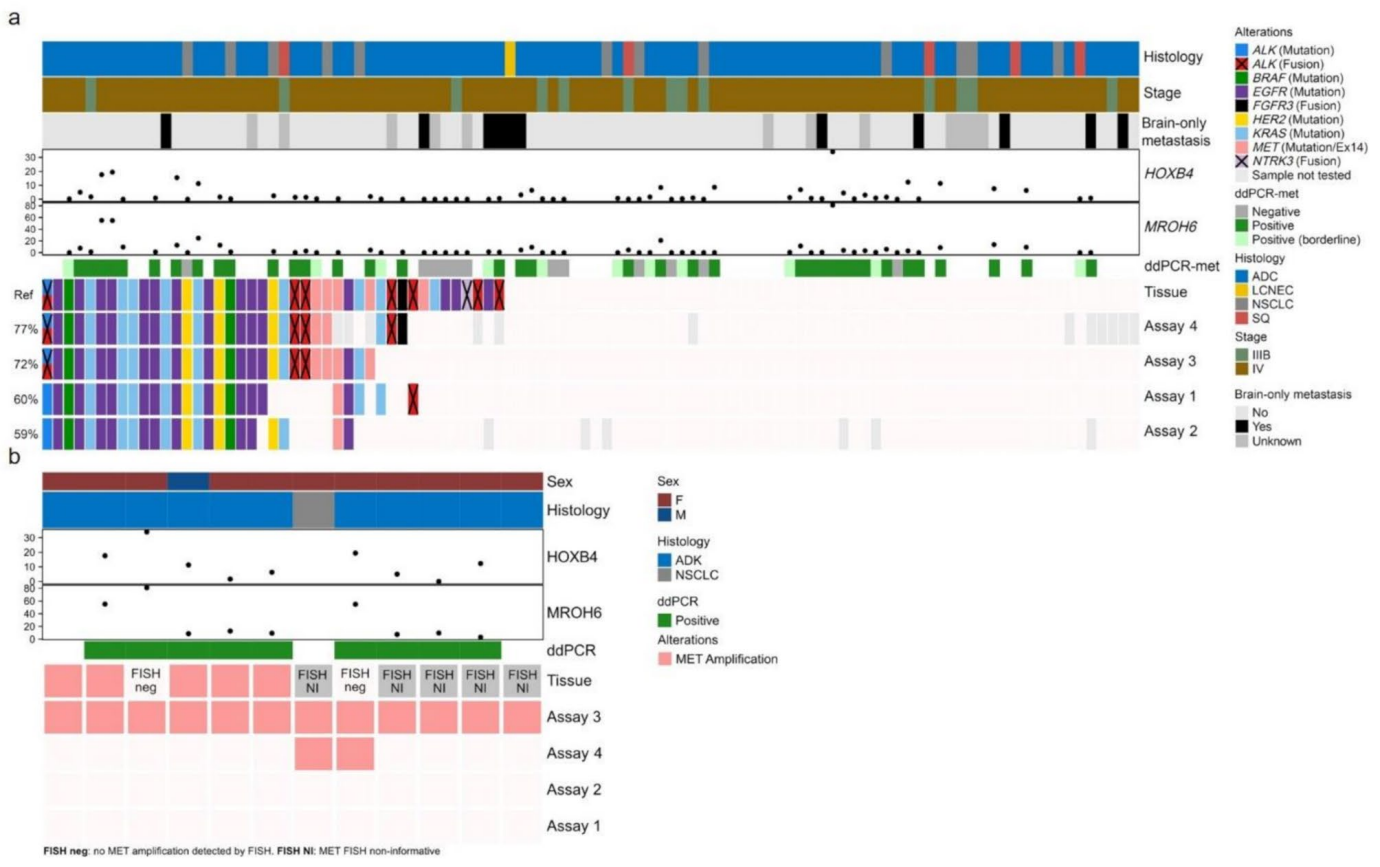
(**a**) Comparison of the detection of ESCAT I/II mutations across all panels and tissue testing. Baseline characteristics as well as the results from ddPCR for both of the two markers (*HOXB4* and *MROH6*) are shown. Concordance of panels with tissue testing is shown in percent to the left. Tissue was used as reference and percent of patients with detected mutations are shown for each panel. (**b**) Oncoprint of *MET* amplifications. Baseline characteristics as well as results from ddPCR for both of the two markers (*HOXB4* and *MROH6*) are shown. FISH neg: Sample was negative for *MET* amplification in tissue FISH testing. FISH NI: FISH sample was non informative in tissue *MET* amplification testing

Figure 5a highlights the results of the methylation status and the concordance between TB-based NGS and LB-based NGS assays. On the one hand, amplicon-based NGS assays (Assays 1 and 2) demonstrated limited efficiency in detecting gene fusions compared to the results of the TB. Assay 1 identified only two gene fusions (one *ALK* fusion and one *MET* exon 14 skipping) and assay 2 identified one *MET* exon 14 skipping. On the other hand, the hybrid capture-based assays (Assays 3 and 4) detected seven gene fusions each. However, TB-based NGS identified a total of 14 gene fusions, indicating incomplete overlap between these approaches (Fig. 5a). The analysis also found that five out of six samples labeled as negative for cfDNA ddPCR-met corresponded to WT results across all cfDNA NGS assays (discordant cases). The one false-negative ddPCR-met sample refers to a *HER2* V777L SNV detected across all NGS assays with a VAF < 1% (Fig. 5a). One positive ddPCR-met sample showed an *ALK* fusion in TB but none of the three LB assays (sample not tested in assay 4) detected the fusion. However, the patient had a brain-only metastasis and low concentration of circulating ddPCR-met biomarkers. Supplementary Figure S5 illustrates the association between the ddPCR-met status and the detection of alterations by significantly showing a higher VAF in cases with a positive ddPCR-met status across all panels. Additionally, we observed significant correlation of the mean VAF for ESCAT I/II mutations across all panels with the ddPCR results for *HOXB4* and *MROH6* (Supplementary Figure S6). Taken together, these data indicate that the ddPCR-met analysis can be a valuable tool in assessing TF and complementing LB data to provide complete genomic profiling, particularly when cfDNA NGS results are WT.

### MET amplification detection

Regarding *MET* CNV amplifications, Assay 3 stood out by detecting 12 *MET* amplifications that were not identified using TB-based NGS. Notably, seven out of these 12 cases were tested with FISH *MET*, and five fulfilled the criteria for amplification (Table 3; Supplementary Figure S7). Two of the seven cases had *MET* CNVs but insufficient amplification, defined as *MET* signal ≥ 6 [40, 41, 46], underscoring the reliability of this specific panel’s results. It highlights the added value of hybrid capture-based panels in scenarios where TB-based NGS may not be comprehensive enough (Fig. 5b).

**Table 3:** Patient characteristics with high-level *MET* amplification by FISH analysis

ID	MET signal/tumour cells, mean, *n*	Ratio MET/CEP7	Genomic results	MET status interpretation [37, 38]
*MET* patient #1	5	1	polysomy	Intermediate level of gene copy number gain
*MET* patient #2	7,31	0,88	polysomy	Amplification
*MET* patient #3	6,7	1,06	polysomy	Amplification
*MET* patient #4	6,8	1,88	polysomy	Amplification
*MET* patient #5	6	1,07	polysomy	Amplification
*MET* patient #6	6,14	1,16	polysomy	Amplification
*MET* patient #7	4,06	1,17	polysomy	Low level of gene copy number gain

CEP7, Centromere enumeration probe 7; FISH, fluorescence in situ hybridisation; *MET*, mesenchymal-epithelial transition gene

### cfDNA data and association with clinical data and overall survival

Significant associations emerged with patient characteristics. Stage IIIB patients and those with brain-only metastases were more likely to show negative cfDNA ddPCR-met results (Supplementary Figure S8a-c). 14.29% of patients with brain-only metastases had negative cfDNA ddPCR-met *versus* 18.37% of patients with no brain-only metastasis (*p* = 1) (Supplementary Figure S8d-f). Brain-only metastatic patients had significant lower concentrations of cfDNA ddPCR-met biomarkers, suggesting that certain disease stages and metastatic profiles may influence the detectability of genetic alterations in LB methods (Supplementary Figure S8d-f). We then assessed the performance of each LB NGS assay stratified by disease stage and “brain-only metastasis” status. Notably, PPA was lower for three LB NGS assays in stage IIIB patients (range: 33–67%) compared to stage IV patients (range: 55–78%), and for patients with brain-only metastases (range: 25–33%) compared to patients with no brain-only metastases (range: 60–84%) (Supplementary table S1 and supplementary table S2).

To study whether ddPCR-met high or low number of copies/20µl is correlated with poor survival in NSCLC patients, we carried out survival analyses based on *HOXB4* and *MROH6* biomarkers. We found that patients with stage IIIB aNSCLC were significantly more likely to have a lower concentration of cfDNA ddPCR-met and better overall survival than stage IV aNSCLC patients, regardless of the biomarker used for analysis (Supplementary Figure S9a, b). Additionally, positive ddPCR-met patients and discordant cases seem to have a lower overall survival (*p* = 0.06 and *p* = 0.077, respectively) (Supplementary Figure S9c, d).

## Discussion

This study provides invaluable insight into the clinical relevance, feasibility, and comparative performance of TB and LB-based NGS methods performed on site in a single hospital centre for genomic profiling of aNSCLC. By evaluating four cfDNA NGS assays—two amplicon-based and two hybrid capture-based—against matched tissue FFPE amplicon-based NGS assays as the gold standard, we highlight their distinct strengths, limitations, and applications in routine clinical practice.

Amplicon-based assays (Assays 1 and 2) offer faster turnaround times and require less DNA input, which is beneficial for low-yield samples. However, their ability to detect complex alterations, such as gene fusions and *MET* amplifications, is more limited. Hybrid capture-based assays (Assays 3 and 4) demonstrated superior accuracy in identifying these genomic alterations, particularly when the ctDNA input exceeded 30 ng. Notably, Assay 3 demonstrated a strong capacity to detect *MET* amplifications confirmed by FISH, while Assay 4 performed better in identifying gene fusions. These findings underscore the importance of selecting assays aligned with sample quantity and clinical objectives.

It is important to highlight that our laboratory operates under unique conditions, which are quite different from standard practices in some other settings [47]. Our TB workload is unusual because we utilise ultrafast NGS with the Thermo Fisher GENEXUS Oncomine Precision Assay (OPA) platform. This means that we observe quite limited clinical relevance with a LB to obtain fast results in our setting [47]. However, in many other laboratory settings, the NGS results with a TB can take up to several weeks to process, making LB more relevant due to its faster turnaround time. Additionally, we benefit from on-site NGS capabilities, whereas in cases where NGS is outsourced, LB NGS tends to be quicker than TB NGS [47].

Moreover, while hybrid capture-based assays have demonstrated greater effectiveness than amplicon-based methods to detect complex genomic alterations in other cancer indications [48], we specifically focused on ESCAT I/II gene alterations in this study. However, it is plausible that comprehensive genomic profiling assays, i.e., NGS assays covering at least 100 genes, identifying ESCAT grade III/IV alterations could be more relevant in aNSCLC [49]. For example, baseline detection of *STK11*, *KEAP1*, *RB1*, and *SMARCA4* may warrant future investigation as their role in disease progression and resistance mechanisms becomes better understood [50–52]. Assay 3, which has the panel with the highest number of genes, can detect numerous ESCAT III/IV alterations that can be useful for inclusion into clinical trials after treatment resistance/relapse but nowadays not at diagnosis.

A high level of concordance was observed across all cfDNA NGS assays in detecting ESCAT I/II SNVs, demonstrating their reliability in identifying point mutations. Agreement was lower for gene fusions and *MET* amplifications, particularly with amplicon-based assays. This highlights the value of TB as the primary diagnostic tool for NGS at tumour diagnosis of aNSCLC. The challenges concerning the cost and reimbursement significantly hinder widespread LB adoption in routine clinical practice, with access varying widely across countries due to inconsistent welfare security and complex approval processes [53]. These issues limit patient access and discourage innovation. Yet, in the real-world scenarios, more than one-third (up to 40%) of traditional TB samples are insufficient or non-viable [9–13], necessitating additional procedures with associated costs and delays. Integrating LB cfDNA analysis into diagnostic workflows can offer significant value by reducing the risk of resampling and incorrect treatment decisions, saving both time and budget.

In our study, the addition of ddPCR-met analysis enhances the utility of LB, particularly in clarifying WT results from cfDNA NGS. For instance, five out of six cfDNA samples deemed negative for ddPCR-met were WT across all cfDNA assays in our study, demonstrating its complementary diagnostic role.

We also showed a correlation between the tumour burden and ctDNA presence/concentration, which was associated with reduced performance of LB NGS assays. A higher tumour burden was associated with increased ctDNA, while stage IIIB or brain-only metastatic patients showed lower ctDNA concentrations, resulting in higher WT rates. These findings highlight the limitations of relying solely on LB in specific patient subgroups and underscore the benefit of integrating both TB and LB methodologies to minimise the risk of false-negative LB results.

Insight from Stetson et al.. highlight the technical variability among NGS assays as a key driver of discordance [54]. False-positive and false-negative variant calls were most common at low VAFs, often below 1%, with variability in sensitivity (38-89%) and PPV (36-80%) across assays [54]. Our findings are consistent with this study, showing similar challenges with assay inaccuracies, especially in detecting low-frequency variants and complex alterations. While hybrid capture-based assays proved more effective than amplicon-based methods, they still did not fully align with the TB results. Similarly, it is important to note that the only false-negative ddPCR-met sample was observed in a case with a low SNV VAF reported across all assays, underscoring the potential performance limitations of this method in cases with low TF/VAF alterations.

Stetson et al.. emphasise that caution is warranted when interpreting plasma-detected variants below 1% VAF due to the high likelihood of false-positives [54]. Likewise, our study demonstrates that the incremental value of LB-first reflex testing is highly assay-dependent, ranging from 0 to 43% across NGS assays, only due to the detection of a *MET* amplification. While Assay 4 achieved the highest accuracy and Predictive Percent Agreement, even it failed to detect some actionable alterations verified by TB NGS and a ddPCR-met analysis. Our results are consistent with a recent study by Bote-de Cabo et al.. that describe in a large cohort of patients with treatment-naïve aNSCLC and matched TB- and LB-based NGS that the addition of orthogonal liquid to tissue biopsy offered no relevant clinical value in cases with ESCAT I or II targetable drivers already detected by TB [55].

Taken together, these data highlight the clinical risk of over-reliance on a single-platform LB-first strategy, especially in cases of low tumour burden or challenging ctDNA conditions observed in specific patient subsets like stage IIIB or brain-only metastases.

To mitigate discordance, platforms with robust performance in detecting low-frequency variants should be prioritised. Incorporating complementary methodologies, such as ddPCR-met, can resolve ambiguities in the results of WT plasma NGS and improve accuracy. Enhancing bioinformatic pipelines, adopting stricter mutational bias filtering, and implementing uniform reporting standards for low-VAF variants are necessary steps to minimise false-positive results. Optimising vendor practices and harmonising quality control will further reduce errors, as noted by Stetson et al. [54].

Overall, our findings support using hybrid capture assays for LB NGS, specifically where an adequate ctDNA input is available, thus complementing TB NGS. Based on the data obtained in this study, our algorithm implements LB NGS as reflex testing when TB NGS fails as a complementary test. However, when there is an urgent clinical need or on demand, LB NGS can be performed first without waiting for the results of TB NGS. Therefore, adopting plasma reflex testing for efficient upfront strategies in aNSCLC should not be done systematically in any cases, according to the results of TB NGS. However, establishing on-site NGS workflows for both TB and LB reduces turnaround times, adding significant value to precision oncology programs. The testing algorithm at tumour progression can be certainly different since doing systematically matched TB and LB NGS can improve the chance to identify an actionable genomic alteration and to detect mechanisms of resistance.

However, our study holds a few limitations. The initial goal of the study was to compare the performance of 4 LB NGS assays on the same blood specimens and then to compare the different results to the TB NGS, which is used as a gold standard in our laboratory. The results did not correlate with clinical outcomes and patient follow-up, and the potential impact of detecting some genomic alterations in LB, notably some additional *MET* genomic alterations, for any treatment decision-making was not available. Then, the TB NGS was based only on an amplicon-based assay, considering that this approach was the gold standard used routinely in our laboratory. In this context, the results of the 4 LB NGS assays were not compared to TB NGS based on hybrid capture technology. Another limitation of this study lies in the inconsistency of quality control measures implemented across the different NGS assays evaluated from cfDNA. Therefore, while positive and negative controls were included as part of the routine diagnostic workflow (Assay 1), no batch-level positive/negative controls were applied for Assay 2, and Assays 3 and 4 included a single positive control only during the first analytical run. The absence of systematic, assay-specific controls across all batches may undermine the interpretability of some discordance analyses, particularly when evaluating assay performance and reproducibility. So, this limitation should be considered when interpreting the comparative results, since potential technical variability cannot be entirely excluded. Finally, the number of patients in this series was limited and heterogeneous since including not only lung adenocarcinoma, but also other NSCLC histological subtypes, and these cases were not all stage IV diseases, some also corresponded to stage III diseases. Moreover, one bias of this study was the inclusion of LB NGS assays, only if matched TB was of “good” quality, meaning with enough quantity and quality of extracted nucleic acid to avoid TB NGS failure, which is not always the case in real-life. So, for this reason, the real added value to perform both TB and LB NGS at diagnosis, including the turnaround times to obtaining the final genomic status, was not totally estimated due to the selection of the cases.

## Conclusion

This retrospective study reinforces the complementary roles of TB and LB in aNSCLC molecular diagnostics, notably at tumour diagnosis, providing a pathway toward improved assay selection and integration. However, we strongly believe that TB is still the gold standard approach for predictive biomarker assessment at baseline, notably when using a NGS assay. LB NGS is of added value at diagnosis of aNSCLC only if TB NGS is not available or failed. By addressing technical discordance between different LB NGS assays, we can enhance the reliability and impact of LB in targeted therapy according to the methodologies. Finally, we showed that using ddPCR for circulating DNA methylation assessment could be very helpful to eliminate the diagnosis of a LB NGS false-negative result.

## Electronic supplementary material

Below is the link to the electronic supplementary material.

Supplementary Material 1

Supplementary Material 2

## Data Availability

The data that support the findings of this study are not openly available due to reasons of sensitivity and are available from the corresponding author upon reasonable request. Data are located in controlled access data storage at Laboratory of Clinical and Experimental Pathology (IHU RespirERA, CHU Nice, Nice, France).

## Abbreviations

ALK: Anaplastic lymphoma kinase
BRAF: B-raf proto-oncogene
cfDNA: Circulating free DNA
CNVs: Copy number variations
ctDNA: Circulating tumour DNA
ddPCR: Droplet digital PCR
EGFR: Epidermal growth factor receptor
ESCAT: ESMO Scale for Clinical Actionability of molecular Targets
ESMO: European Society for Medical Oncology
FFPE: Formalin-fixed paraffin-embedded
FISH: Fluorescence in situ hybridisation
LB: Liquid biopsy
MET: Mesenchymal epithelial transition
NGS: Next-generation sequencing
NPA: Negative percent agreement
NPV: Negative predictive value
NSCLC: Non-small cell lung carcinoma
PCR: Polymerase chain reaction
PPA: Positive percent agreement
PPV: Positive predictive value
RET: Rearranged during transfection
ROI: Region of interest
ROS1: ROS proto-oncogene 1
SNV: Single-nucleotide variant
TB: Tissue biopsy
TF: Tumour fraction
UMI: Unique molecular identifier
VAF: Variant allele frequency
WT: Wild-type
