# Supplementary material for: Detecting actionable mutations from matched plasma-based versus tissue next-generation sequencing in advanced non-small cell lung cancer: a retrospective single centre analysis on site
Source: J Exp Clin Cancer Res. 2025 Aug 6;44:229. doi: 10.1186/s13046-025-03480-x (PMC12326616; doi:10.1186/s13046-025-03480-x)
Supplement: Supplementary file 1 — Supplementary Material 1 [file 13046_2025_3480_MOESM1_ESM.doc]

**DETECTING ACTIONABLE MUTATIONS FROM MATCHED PLASMA-BASED VERSUS TISSUE NEXT-GENERATION SEQUENCING IN ADVANCED NON-SMALL CELL LUNG CANCER: A RETROSPECTIVE SINGLE CENTRE ANALYSIS ON SITE**

Christophe Bontoux^1-6†^, Caroline Lacoux^3-6†^, Jonathan Benzaquen^3,6,7,8^, Jacques Boutros^3,7,8^, Guylène Rignol^3-6,8^, Elodie Long-Mira^3-6,8^, Sandra Lassalle^3-6^, Maryline Allegra^3-6,8^, Doriane Bohly^3-6^, Mathieu Garcia^3-6^, Christelle Bonnetaud^3-6^, Olivier Bordone^3-6^, Jean-Marc Félix^3-6^, Virginie Lespinet-Fabre^3-6^, Virginie Tanga^3-6^, Charles-Hugo Marquette^3,6,7,8^, Valérie Taly^9^, Aurélia Baurès^10^, Simon Heeke^11^, Marius Ilié^3-6,8^, Véronique Hofman^3-6,8^ and Paul Hofman^3-6,8^*

^1^ Department of Pathology, Cancer University Institute of Toulouse-Oncopole, University Hospital of Toulouse, 31059 Toulouse, France.

^2^ OncoSarc, INSERM U1037, Cancer Research Center in Toulouse, 31000 Toulouse, France.

^3^ Institut Hospitalo-Universitaire RespirERA, Université Côte d’Azur, Hôpital Pasteur, CHU de Nice, 06001 Nice CEDEX 1, France.

^4^ Laboratory of Clinical and Experimental Pathology, Université Côte d’Azur, Hôpital Pasteur, CHU de Nice, France.

^5^ Hospital-Integrated Biobank (BB-0033-00025), Université Côte d’Azur, Hôpital Pasteur, CHU de Nice, 06001 Nice CEDEX 1, France.

^6^ FHU OncoAge, Université Côte d’Azur, 06001 Nice CEDEX 1, France.

^7^ Université Côte d’Azur, CHU Nice, FHU OncoAge, IHU respirERA, Department of Pneumology, Pasteur Hospital, Nice, France.

^8^ Team 4, Institute of Research on Cancer and Aging of Nice (IRCAN), Inserm U1081, CNRS UMR7284, Université Côte d’Azur, CHU de Nice, 06107 Nice CEDEX 2, France.

^9^ Université de Paris, UMR-S1138, CNRS SNC5096, Équipe Labélisée Ligue Nationale Contre le Cancer, Centre de Recherche des Cordeliers, Paris, France.

^10^ METHYS Dx, 67 rue Saint-Jacques, 75005 Paris, France.

^11^ Department of Thoracic/Head & Neck Medical Oncology, The University of Texas MD Anderson Cancer Center, Houston, TX, USA.

^†^These authors contributed equally to this work

***Corresponding author:**

Pr. Paul Hofman, MD, PhD

Laboratory of Clinical and Experimental Pathology, Université Côte d’Azur, Hôpital Pasteur, CHU de Nice, 06000, 30 Voie Romaine, Nice, France.

Email: [hofman.p@chu-nice.fr](mailto:hofman.p@chu-nice.fr)

**SUPPLEMENTARY INFORMATION**

**Supplementary Figure S1**: Workflows of cfDNA NGS assays.

**Supplementary Figure S2**: Oncoprint showing all detected mutations across all panels and tissue testing. The total number of detected mutations is shown above the plot. Baseline characteristics as well as results from ddPCR for both of the two markers (*HOXB4* and *MROH6*) are shown. Percentages with the fraction of patients with mutations in the respective gene are shown.

**Supplementary figure S3**: Starting material impact on ESCAT activable Fusion, CNV detection (a) and SNV detection (b). For each alteration type a dot plot correlated to the alteration detected (True positive) or no detection (False-Positive) are shown on the right. On the left side, a Whisker scatter-plot illustrates the range of starting material used for each assay for the corresponding alteration types. The red dotted line represents the 30ng input threshold.

**Supplementary Figure S4.** VAF (SNV), fold-change (CNV; scoring for CNV are differently recording for Avenio and Hedera panels respectively) and number of reads (only for fusions) variations across assays. The red dotted line represents the 1% threshold.

**Supplementary Figure S5.** Mean VAF for ESCAT I/II mutations across all panels for ddPCR-met outcome. P-value for comparison across groups shown above for each of the four assays.

**Supplementary Figure S6**. Correlation of the mean VAF for ESCAT1/2 mutations across all panels with the ddPCR results for *HOXB4* (upper panel) and *MROH6* (lower panel). The coefficient of determination (R) as well as the p-value are shown.

**Supplementary Figure S7**: *MET* FISH assay showing four cases with a high copy number of the MET gene (average copy number ≥6.0 per tumour cell considered as amplification). Red spots represent the mesenchymal-epithelial transition (MET) gene, green spots represent Centromere enumeration probe 7 (CEP7), and nuclei are stained with DAPI (4′,6-diamidino-2-phenylindole; blue). A – MET patient #2, tissue section (oil immersion, x100); B – MET patient #3, tissue section (oil immersion, x60); C – MET patient #4, cytology specimen (oil immersion, x100); and D – MET patient #6, cytology specimen (oil immersion, x60).

**Supplementary Figure S8**: Box plots depicting *HOXB4* (a) and *MROH6* (b) cfDNA ddPCR-met biomarker concentration according to the brain metastatic status. (c) Primary tumour size and cfDNA ddPCR-met results according to the brain-only metastatic status. Box plots depicting primary tumor size (d) and stage (e) according to cfDNA ddPCR-met status. (f) Clinical features according to the cfDNA ddPCR-met status.

**Supplementary Figure S9.** Kaplan-Meier probabilities of overall survival for NSCLC patients according to the status of the two biomarkers used in the cfDNA ddPCR-met analysis (a) *HOXB4* and (b) *MROH6*, and according to (c) the ddPCR-met status Univariate log-rank analysis.

**Supplementary table S1.** Performance of each panel assay tested in the study for ESCAT I/II gene alteration detection stratified by stage.

**Supplementary table S2.** Performance of each panel assay tested in the study for ESCAT I/II gene alteration detection stratified by “brain-only metastasis” status.


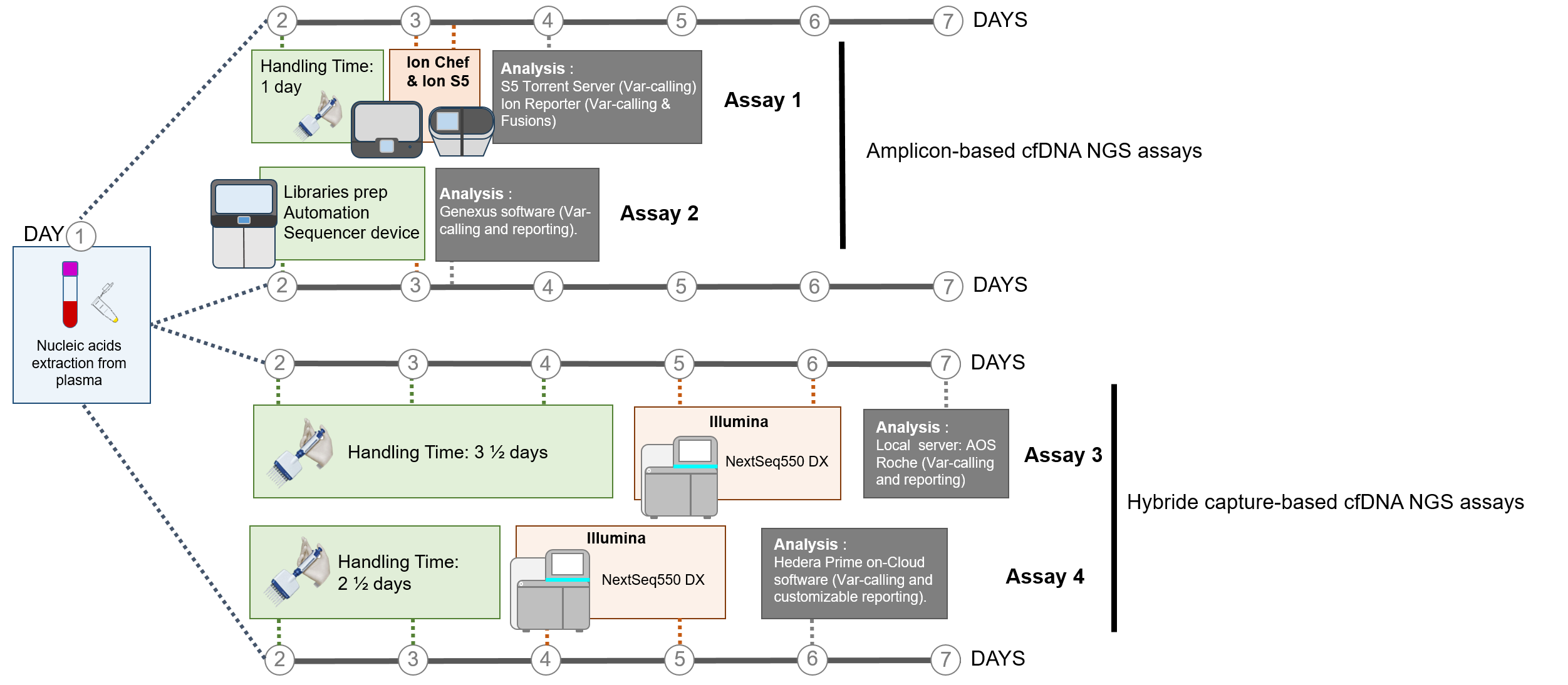


**Supplementary Figure S1**: Workflows of cfDNA NGS assays.


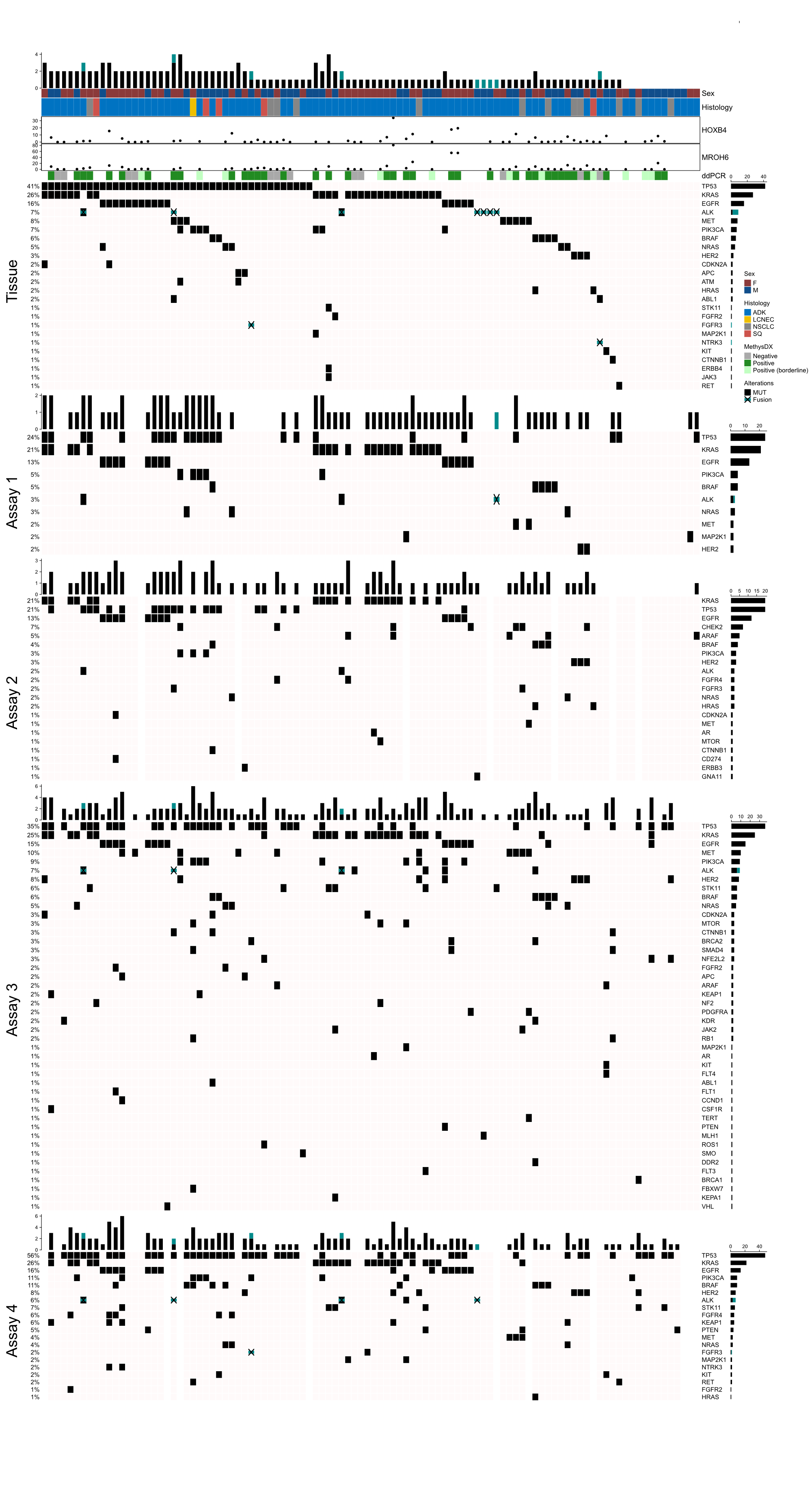


**Supplementary Figure S2**: Distribution of all the gene alterations detected in assays (hybrid capture-based and amplicon-based) for: a) TB samples; b) LB samples using Amplicon-based assays; and c) LB samples using hybrid capture-based assays.

**
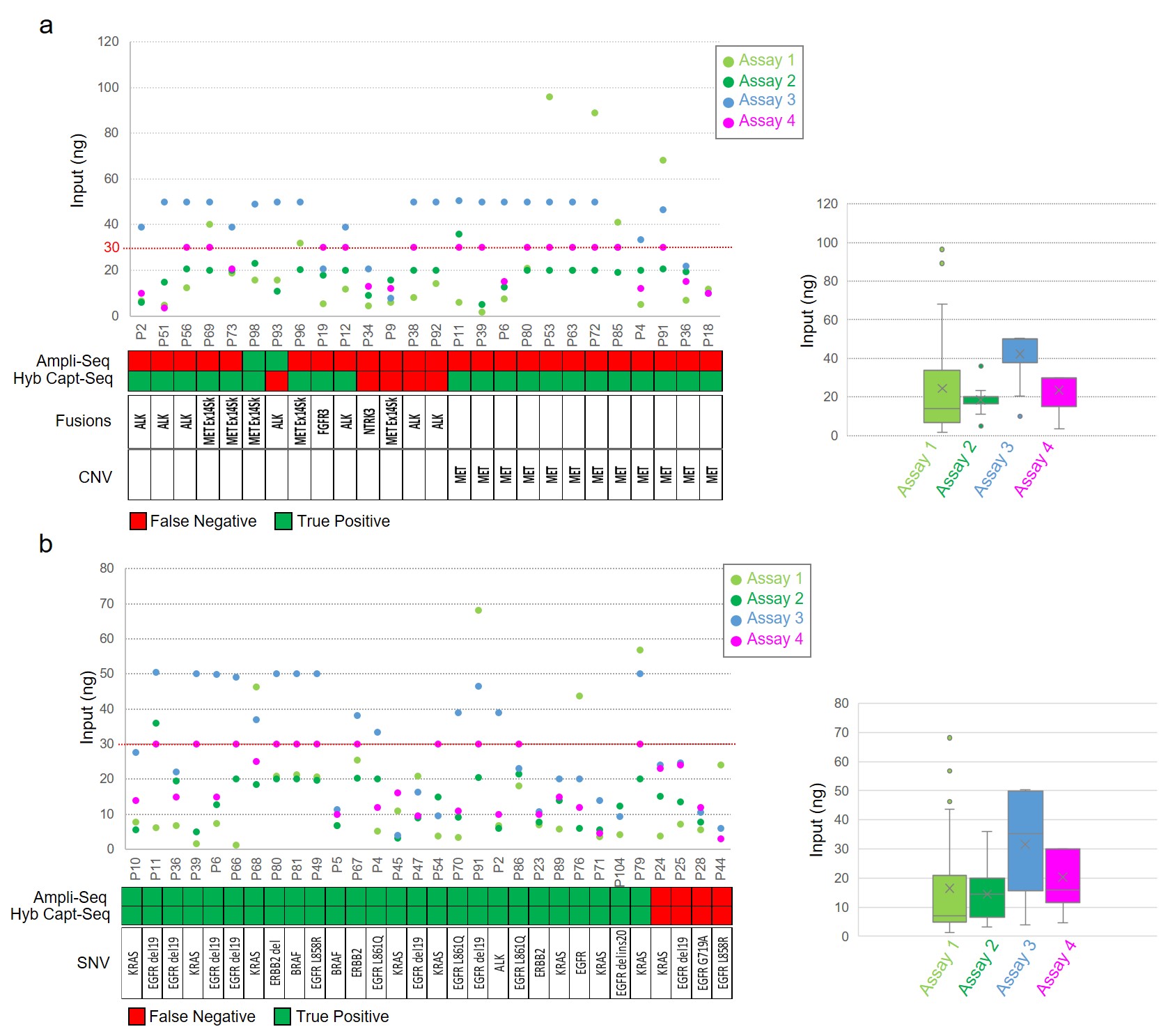
**

**Supplementary figure S3**: Starting material impact on ESCAT activable Fusion, CNV detection (a) and SNV detection (b). For each alteration type a dot plot correlated to the alteration detected (True positive) or no detection (False-Positive) are shown on the right. On the left side, a Whisker scatter-plot illustrates the range of starting material used in each assay for the corresponding alteration type. The red dotted line represents the 30ng input threshold.

**
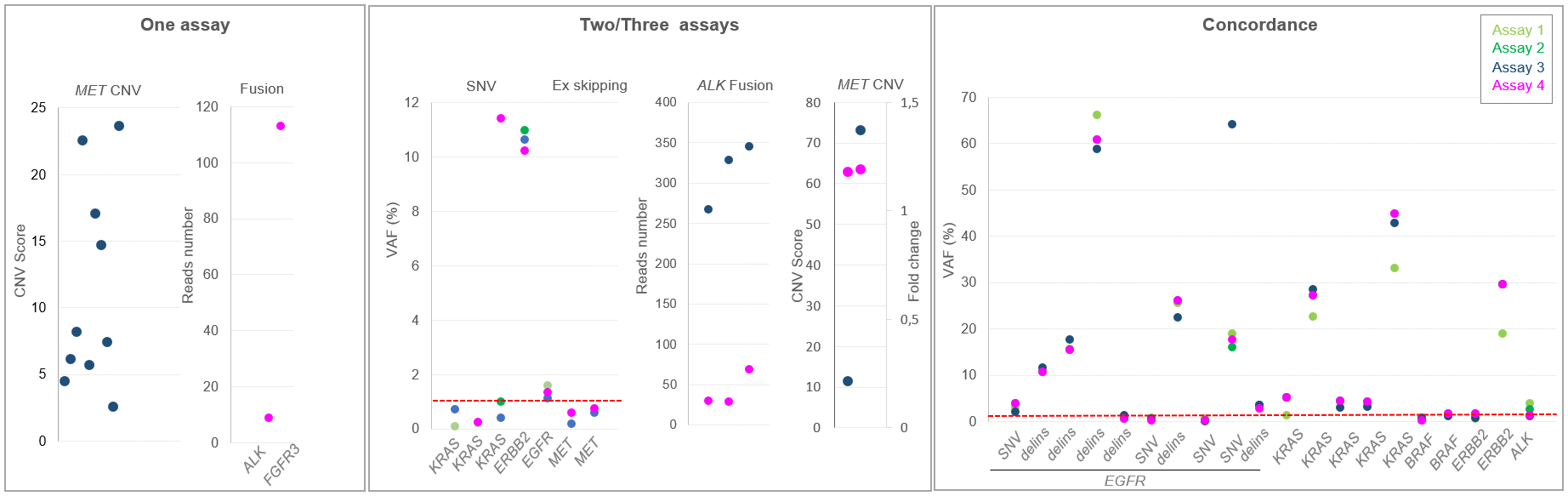

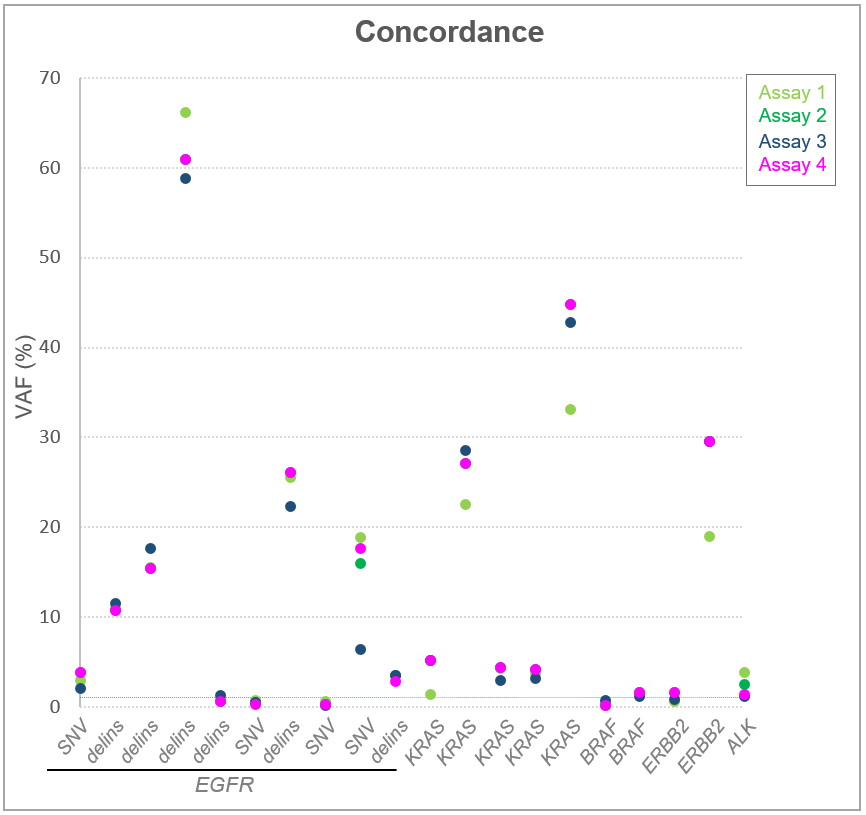
**

**Supplementary Figure S4.** VAF (SNV), fold-change (CNV; scoring for CNV are differently recording for Avenio and Hedera panels respectively) and number of reads (only for fusions) variations across assays. The red dotted line represents the 1% threshold.


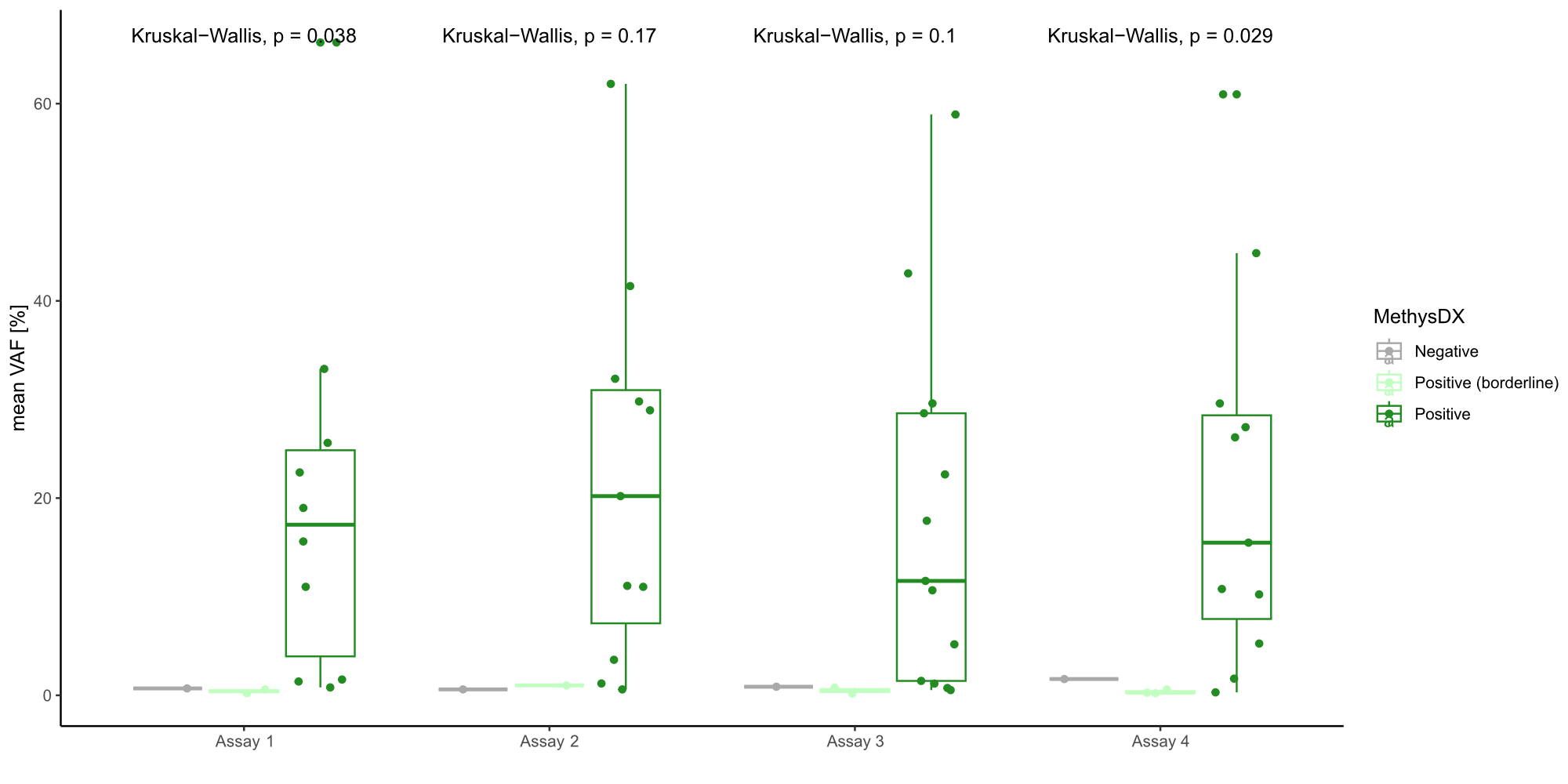


**Supplementary Figure S5.** Mean VAF for ESCAT I/II mutations across all panels for ddPCR-met outcome. P-value for comparison across groups shown above for each of the four assays.

**
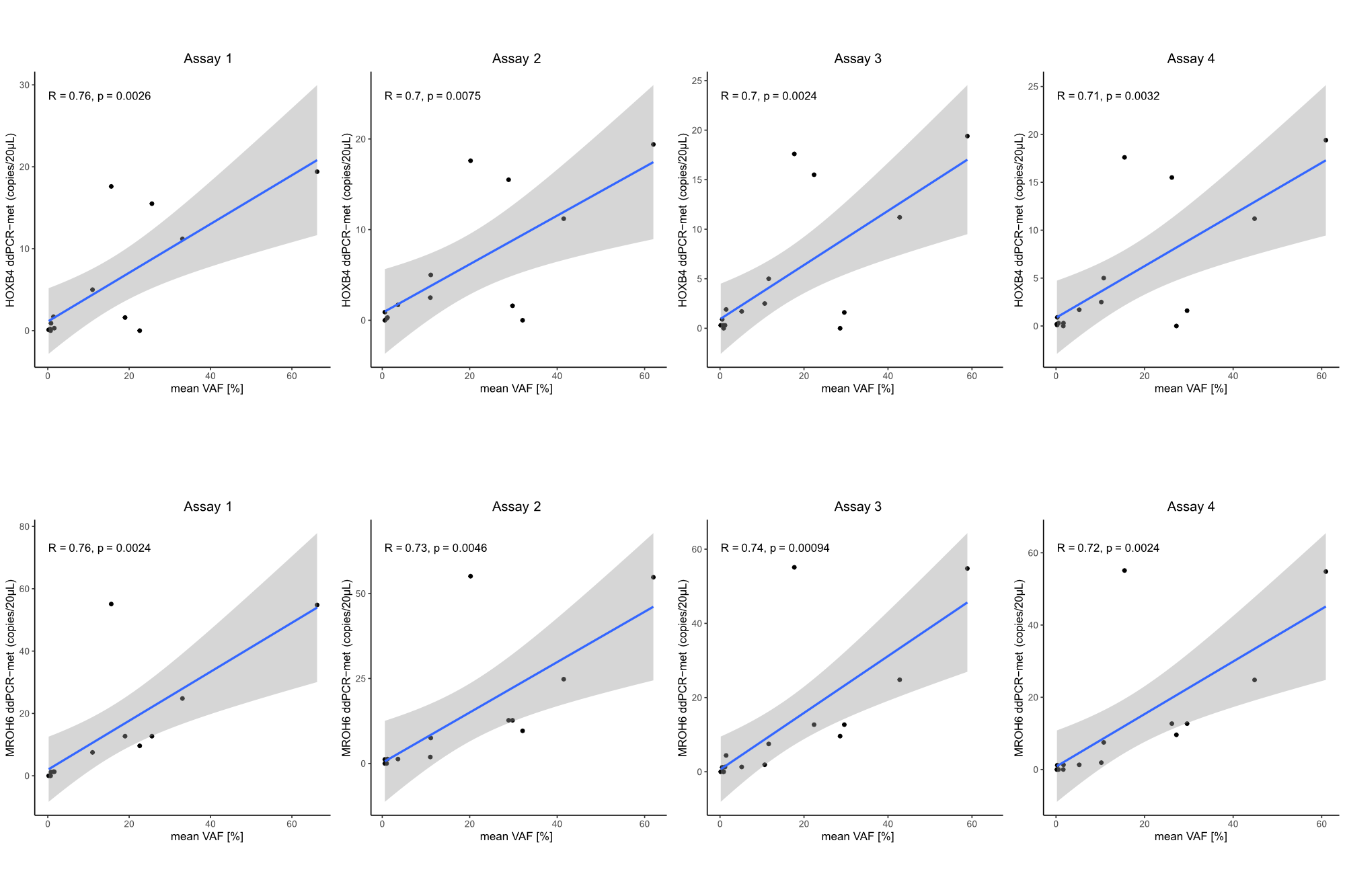
**

**Supplementary Figure S4.** Correlation of variant allele frequency (VAF) for ESCAT1/2 mutations across all panels. Coefficient of determination (R) as well as p-value is shown.

**Supplementary Figure S6**. Correlation of the mean VAF for ESCATI/II mutations across all panels with the ddPCR results for *HOXB4* (upper panel) and *MROH6* (lower panel). The coefficient of determination (R) as well as the p-value are shown.


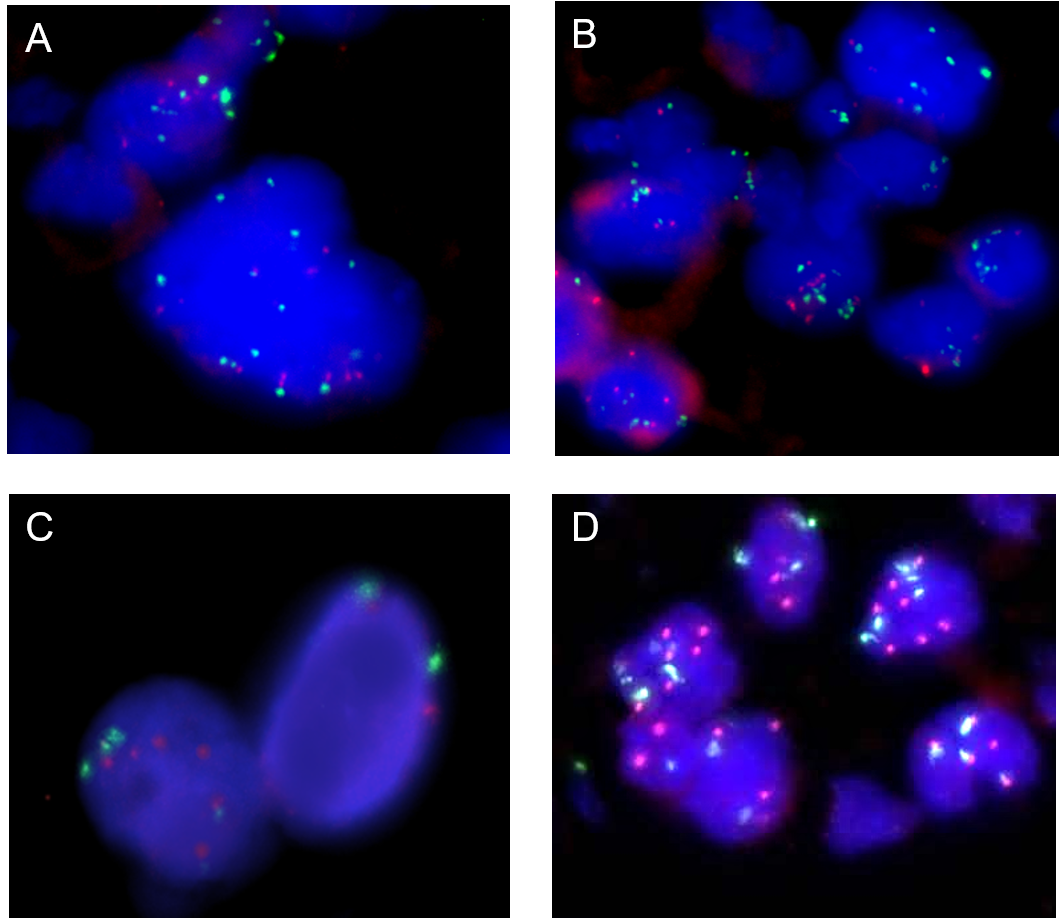


**Supplementary Figure S7**: *MET* FISH assay showing four cases a high copy number of the *MET* gene (average copy number ≥6.0 per tumour cell considered as amplification). Red spots represent the mesenchymal-epithelial transition (MET) gene, green spots represent Centromere enumeration probe 7 (CEP7), and nuclei are stained with DAPI (4′,6-diamidino-2-phenylindole; blue). A – MET patient #2, tissue section (oil immersion, x100); B – MET patient #3, tissue section (oil immersion, x60); C – MET patient #4, cytology specimen (oil immersion, x100); and D – MET patient #6, cytology specimen (oil immersion, x60


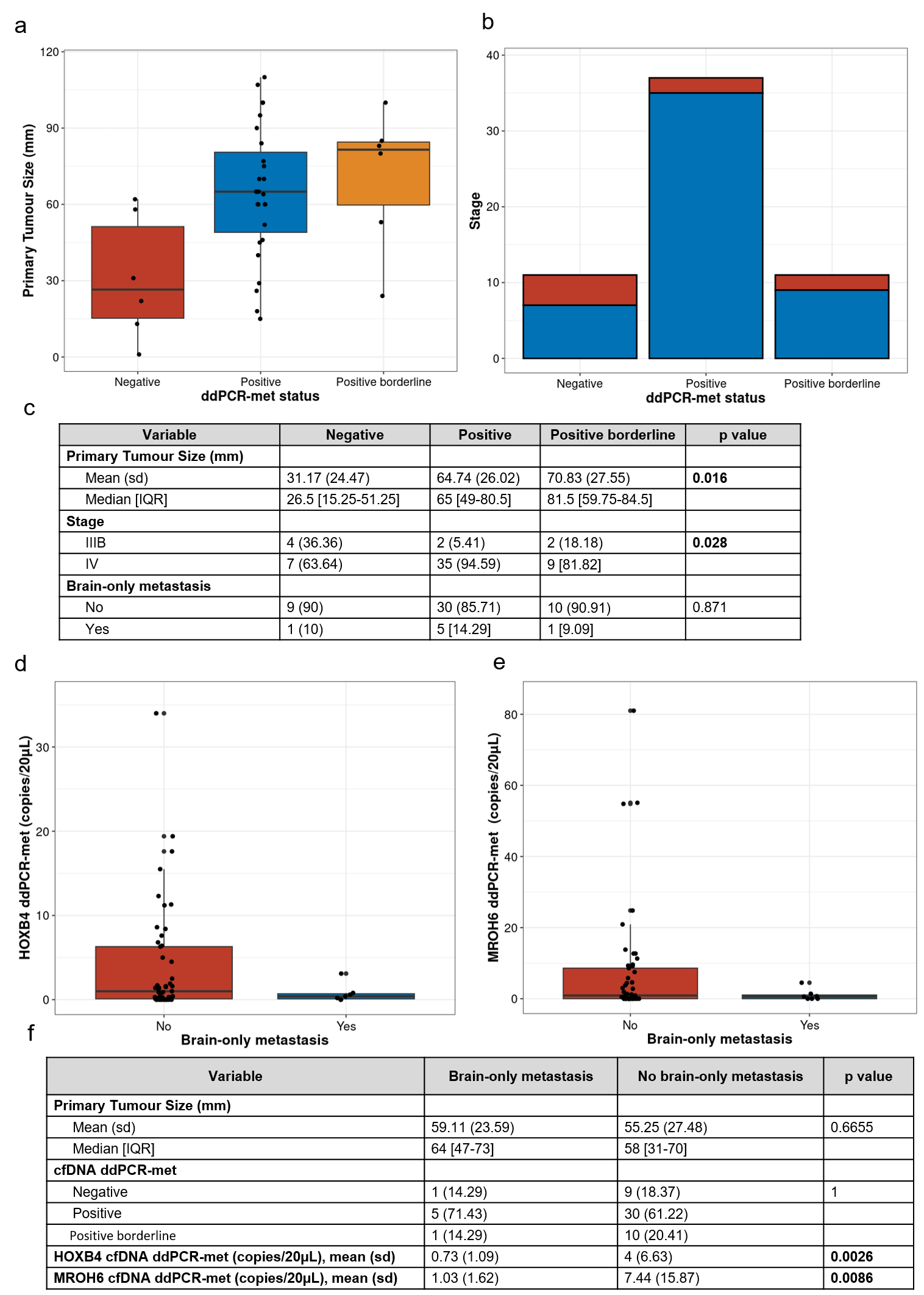

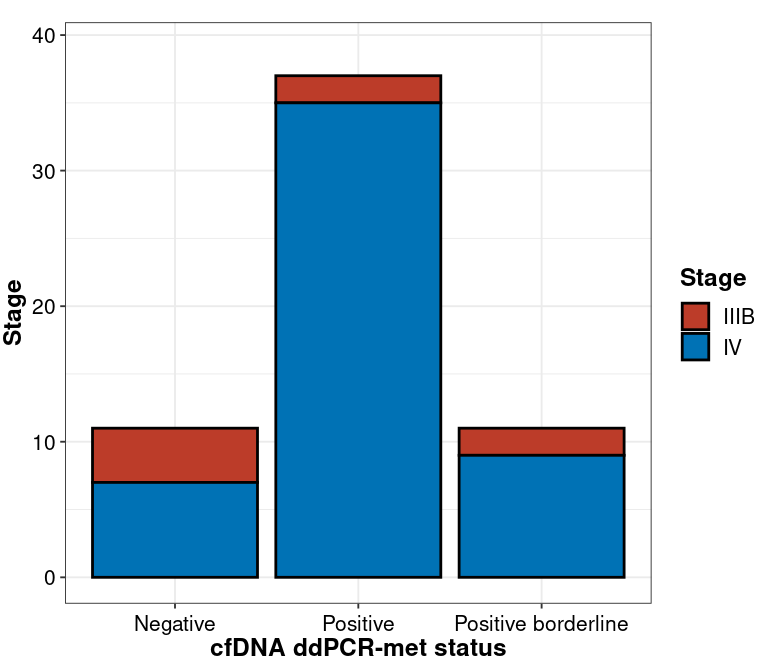


**Supplementary Figure S8**: Box plots depicting the *HOXB4* (a) and *MROH6* (b) cfDNA ddPCR-met biomarker concentration according to the metastatic status. c) Primary tumour size and cfDNA ddPCR-met results according to the brain-only metastatic status. Box plots depicting the primary tumour size (d) and stage (e) according to cfDNA ddPCR-met status. f) Clinical features according to the cfDNA ddPCR-met status.


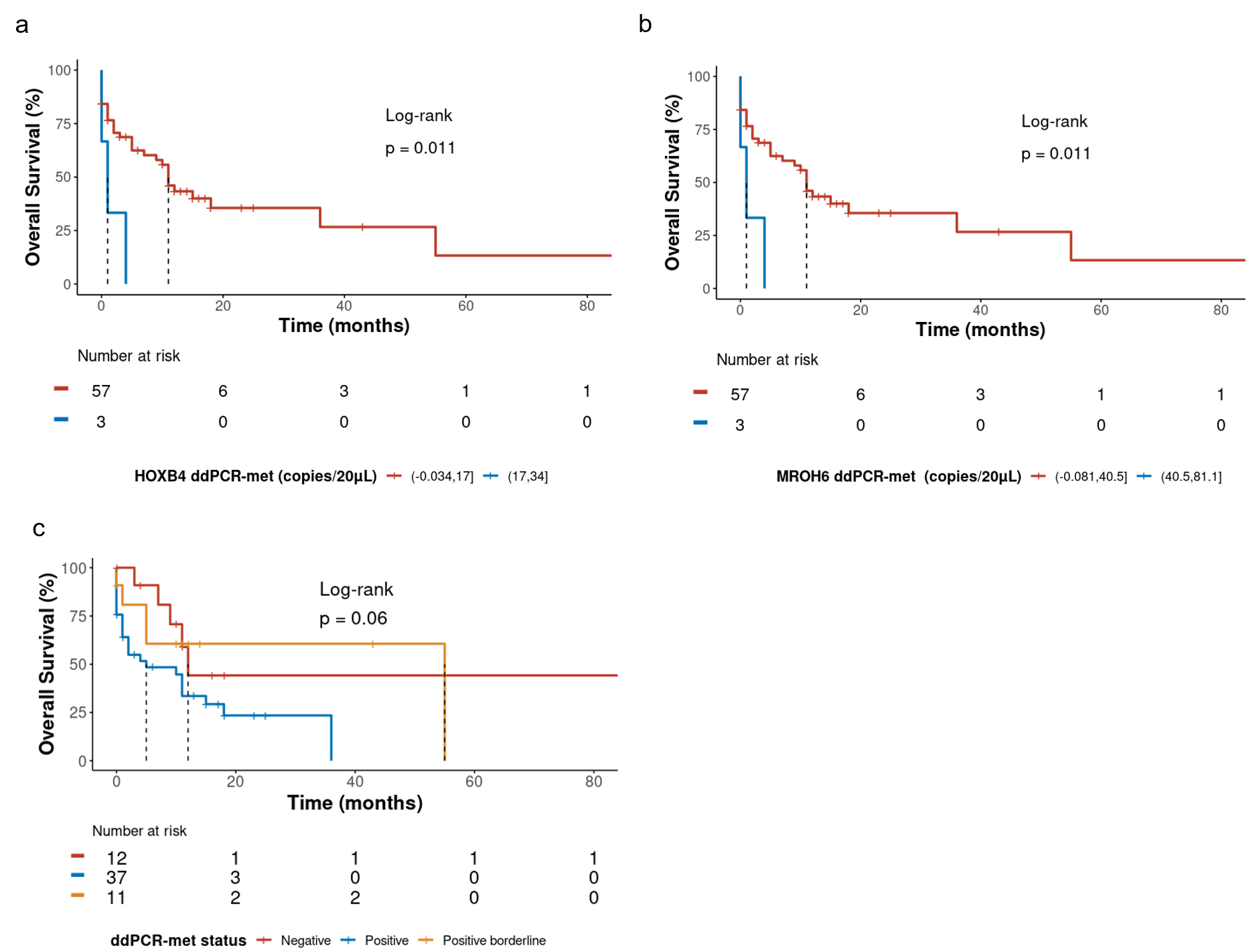


d


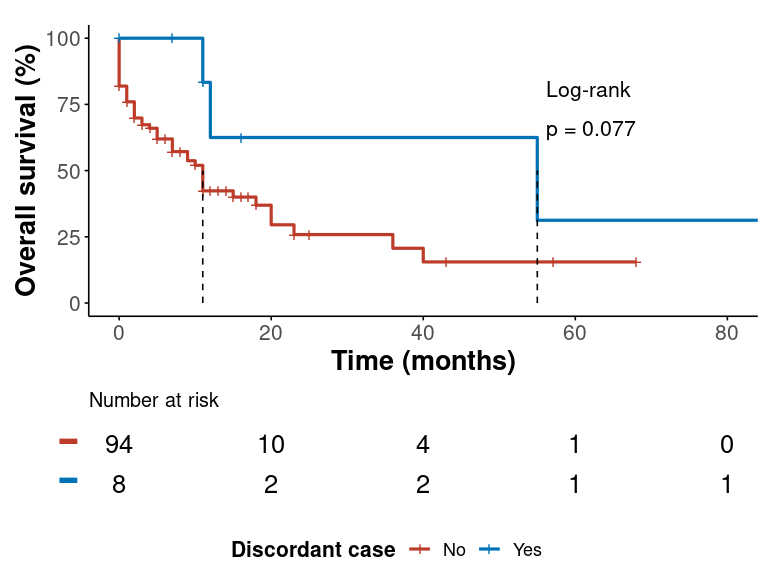


**Supplementary Figure S9.** Kaplan-Meier probabilities of overall survival for NSCLC patients according to the status of the two biomarkers used in the cfDNA ddPCR-met analysis (a) *HOXB4* and (b) *MROH6*, according to (c) the ddPCR-met status Univariate log-rank analysis, and according to (d) the discordant status. Univariate log-rank analysis.

**Supplementary table S1. Performance of each panel assay tested in the study for ESCAT I/II gene alteration detection stratified by stage**

| **ESCAT I/II alterations** **detection** | | | | | | | | |
| --- | --- | --- | --- | --- | --- | --- | --- | --- |
|  | **Stage IIIB Patients, N = 13** | | | | **Stage IV Patients, N = 89** | | | |
|  | **Assay 1 (N=13)** | **Assay 2 (N=13)** | **Assay 3 (N=13)** | **Assay 4 (N=12)** | **Assay 1 (N=89)** | **Assay 2 (N=83)** | **Assay 3 (N=89)** | **Assay 4 (N=78)** |
| **PPA** | 33% | 67% | 67% | 67% | 61% | 55% | 71% | 78% |
| **NPA** | 100% | 100% | 100% | 100% | 100% | 100% | 88% | 98% |
| **Accuracy** | 85% | 92% | 92% | 92% | 82% | 78% | 80% | 88% |
| **PPV** | 100% | 100% | 100% | 100% | 100% | 100% | 83% | 97% |
| **NPV** | 83% | 91% | 91% | 90% | 75% | 70% | 78% | 84% |

ESCAT, ESMO Scale for Clinical Actionability of molecular Targets; PPA, positive percent agreement; mut, mutated; NPA, negative percent agreement; PPV, positive predicting value; NPV, negative predicting value; WT, wild-type.

**Supplementary table S2. Performance of each panel assay tested in the study for ESCAT I/II gene alteration detection stratified by “brain-only metastasis” status**

| **ESCAT I/II alterations** **detection** | | | | | | | | |
| --- | --- | --- | --- | --- | --- | --- | --- | --- |
|  | **Brain-only metastasis patients, N = 11** | | | | **No brain-only metastasis patients, N = 79** | | | |
|  | **Assay 1 (N=11)** | **Assay 2 (N=9)** | **Assay 3 (N=11)** | **Assay 4 (N=8)** | **Assay 1 (N=79)** | **Assay 2 (N=75)** | **Assay 3 (N=79)** | **Assay 4 (N=70)** |
| **PPA** | 25% | 33% | 25% | 33% | 69% | 60% | 80% | 84% |
| **NPA** | 100% | 100% | 100% | 100% | 98% | 100% | 89% | 97% |
| **Accuracy** | 73% | 78% | 73% | 75% | 85% | 82% | 85% | 91% |
| **PPV** | 100% | 100% | 100% | 100% | 96% | 100% | 85% | 96% |
| **NPV** | 70% | 75% | 70% | 71% | 80% | 75% | 85% | 88% |

ESCAT, ESMO Scale for Clinical Actionability of molecular Targets; PPA, positive percent agreement; mut, mutated; NPA, negative percent agreement; PPV, positive predicting value; NPV, negative predicting value; WT, wild-type.
