# Supplementary material for: Detecting actionable mutations from matched plasma-based versus tissue next-generation sequencing in advanced non-small cell lung cancer: a retrospective single centre analysis on site
Source: J Exp Clin Cancer Res. 2025 Aug 6;44:229. doi: 10.1186/s13046-025-03480-x (PMC12326616; doi:10.1186/s13046-025-03480-x)
Supplement: Supplementary file 2 — Supplementary Material 2 [file 13046_2025_3480_MOESM2_ESM.docx]

**DETECTING ACTIONABLE MUTATIONS FROM MATCHED PLASMA-BASED VERSUS TISSUE NEXT-GENERATION SEQUENCING IN ADVANCED NON-SMALL CELL LUNG CANCER: A RETROSPECTIVE SINGLE CENTRE ANALYSIS ON SITE**

Christophe Bontoux^1-6†^, Caroline Lacoux^3-6†^, Jonathan Benzaquen^3,6,7,8^, Jacques Boutros^3,7,8^, Guylène Rignol^3-6,8^, Elodie Long-Mira^3-6,8^, Sandra Lassalle^3-6^, Maryline Allegra^3-6,8^, Doriane Bohly^3-6^, Mathieu Garcia^3-6^, Christelle Bonnetaud^3-6^, Olivier Bordone^3-6^, Jean-Marc Félix^3-6^, Virginie Lespinet-Fabre^3-6^, Virginie Tanga^3-6^, Charles-Hugo Marquette^3,6,7,8^, Valérie Taly^9^, Aurélia Baurès^10^, Simon Heeke^11^, Marius Ilié^3-6,8^, Véronique Hofman^3-6,8^ and Paul Hofman^3-6,8^*

^1^ Department of Pathology, Cancer University Institute of Toulouse-Oncopole, University Hospital of Toulouse, 31059 Toulouse, France.

^2^ OncoSarc, INSERM U1037, Cancer Research Center in Toulouse, 31000 Toulouse, France.

^3^ Institut Hospitalo-Universitaire RespirERA, Université Côte d’Azur, Hôpital Pasteur, CHU de Nice, 06001 Nice CEDEX 1, France.

^4^ Laboratory of Clinical and Experimental Pathology, Université Côte d’Azur, Hôpital Pasteur, CHU de Nice, France.

^5^ Hospital-Integrated Biobank (BB-0033-00025), Université Côte d’Azur, Hôpital Pasteur, CHU de Nice, 06001 Nice CEDEX 1, France.

^6^ FHU OncoAge, Université Côte d’Azur, 06001 Nice CEDEX 1, France.

^7^ Université Côte d’Azur, CHU Nice, FHU OncoAge, IHU respirERA, Department of Pneumology, Pasteur Hospital, Nice, France.

^8^ Team 4, Institute of Research on Cancer and Aging of Nice (IRCAN), Inserm U1081, CNRS UMR7284, Université Côte d’Azur, CHU de Nice, 06107 Nice CEDEX 2, France.

^9^ Université de Paris, UMR-S1138, CNRS SNC5096, Équipe Labélisée Ligue Nationale Contre le Cancer, Centre de Recherche des Cordeliers, Paris, France.

^10^ METHYS Dx, 67 rue Saint-Jacques, 75005 Paris, France.

^11^ Department of Thoracic/Head & Neck Medical Oncology, The University of Texas MD Anderson Cancer Center, Houston, TX, USA.

^†^These authors contributed equally to this work

***Corresponding author:**

Pr. Paul Hofman, MD, PhD

Laboratory of Clinical and Experimental Pathology, Université Côte d’Azur, Hôpital Pasteur, CHU de Nice, 06000, 30 Voie Romaine, Nice, France.

Email: [hofman.p@chu-nice.fr](mailto:hofman.p@chu-nice.fr)

**SUPPLEMENTARY INFORMATION – MATERIALS AND METHODS**

***Description of the Liquid Biopsy NGS assays***

**LBx Assay 1 (Oncomine lung cell free DNA)**

- coverage of hot spots of 11 genes
- 1.8 kb panel size
- genes: exons 21 to 25 for *ALK*; exon 11 and 15 for *BRAF*; exons 18 to 21 for *EGFR*; exon 20 for *ERBB2*; exons 2 to 3 for *KRAS*; exons 2 to 3 and 6 for *MAP2K1*; exon 14, 16 and 19 for *MET*; exons 2 and 3 for *NRAS*; exons 10 and 21 for *PIK3CA*; exon 36 for *ROS1* and exons 4 to 8 and 10 for *TP53*

**LBx Assay 2 (Oncomine Precision Assay-RUO)**

- coverage of 45 genes
- 14.147 kb panel size
- genes: *AKT1* exon 3 to 4; *AKT2* exon 3 and 11; *AKT3* exons 2 to 3; *ALK* exon 2 and 21 to 29; *AR* exon 1 to 8; *ARAF* exon 7; *BRAF* exon 11 and 15; *CD274* exon 1 to 7; *CDK4* exon 2; *CDKN2A* exon 1 to 3; *CHEK2* exon 11 and 15; *CTNNB1* exon 3 and 7; *EGFR* exon 3, 7, 11 to 12, 15, 18 to 21 and 24 to 26; *ERBB2* exon 8 to 9, 12, 17 to 22 and 25; *ERBB3* exon 2 to 4, 7 to 9, 14, 16, 20 and 26; *ERBB4* exon 18; *ESR1* exon 5 to 6 and 8 to 9; *FGFR1* exon 2, 4, 7, 10, 12 to 14; *FGFR2* exon 5, 7 to 9, 12 to 15 and 18; *FGFR3* exon 3, 7, 9, 11 to 16; FGFR4 exon 13; *FLT3* exon 20; *GNA11* exon 4 to 5; *GNAQ* exon 4 to 5; *GNAS* exon 8 to 9; HRAS exon 2 to 3; *IDH1* exon 4 and 6 to 7; *IDH2* exons 4 and 7; *KIT* exon 8 to 11, 13 to 14, 17 to 18; *KRAS* exon 2 to 4, 6; *MAP2K2* exon 2 to 3, *MAP2K1* exon 2 to 3 and 6; *MET* exon 3, 5 to 6, 10, 12, 14, 16; *MTOR* exon 30, 39 to 40, 43, 45, 47, 53; *NRAS* exon 2 to 4; *NTRK1* exon 14 and 15; *NTRK2* exon 18 and 19; *NTRK3* exon 16 and 17; *PDGFRA* exon 11 to 12, 14 and 18; *PIK3CA* exon 2 to 3, 5, 8, 10 to 11, 20 to 21; *PTEN* exon 1 to 2, 4 to 9; *RAF1* exon 7; *RET* exon 10 to 11, 13 to 16; *ROS1* exon 36 to 38; *SMO* exon 6, 8 to 9; *TP53* exon 5 to 8

**LBx Assay 3 (Avenio expanded panel-RUO)**

- coverage of 77 genes
- 200 kb panel size
- All exons of the following genes are covered: *APC, AR, BRCA1, BRCA2, CCND1, CCND2, CCND3, CD274, CDK4, CDKN2A, EGFR, ERBB2, ESR1, FBXW7, KEAP1, KRAS, SMO, STK11, MET, MLH1, MSH2, MSH6, NF2, PDCD1LG2, PMS2, PTEN, RB1, SMAD4, TP53, and VHL.*
- Only some exons and hot spots are covered for the following genes: *ABL1, AKT1, AKT2, ALK, ARAF, BRAF, CDK6, CSFR1, CTNNB1, DDR2, DPYD, EZH2, FGFR1, FGFR2, FGFR3, FLT1, FLT3, FLT4, GATA3, GNA11, GNAQ, GNAS, IDH1, IDH2, JAK2, JAK3, KDR, KIT, MAP2K1, MAP2K2, MTOR, NFE2L2, NRAS, NTRK1, PDGFRA, PDGFRB, PIK3CA, PIK3R1, PTCH1, RAF1, RET, ROS1, RNF43, TERT* prom*, TSC1, TSC2 and UTG1A1.*

**LBx Assay 4 (HP2 Hedera-RUO)**

- coverage of 32 genes
- 90 kb panel size
- All the exons of the following genes are covered: *ALK, BRAF, ERBB2, EGFR, FGFR1, FGFR2, FGFR3, FGFR4, HRAS, KEAP1, KIT, KRAS, MET, NRAS, PIK3CA, PTEN, RET, STK11, TP53.*
- Partial coding DNA sequence (CDS) is covered for the following genes: exon 4 for *AKT1*, exon 5 to 8 for *ESR1*, exon 4 to 5 for *GNA11*, exon 4 and 5 for *GNAQ*, exon 8 to 9 for *GNAS*, Exon4 for *IDH1*, exon 4 for *IDH2*, exon 2 to 6 and exon 8 for *MAP2K1*, exon 8 to 17 for *NTRK1*, exon 14 to 19 for *NTRK2*, exon 15 to 20 for *NTRK3* and exon 32 to 41 for *ROS1*.
